# Digital feedback via free ChatGPT within the reciprocal teaching style: improving fundamental handball skills and students' attitudes among university beginners

**DOI:** 10.3389/fspor.2026.1772502

**Published:** 2026-03-25

**Authors:** Ahmed Hassan Rakha

**Affiliations:** Department of Physical Education and Kinesiology, College of Education, Qassim University, Buraidah, Saudi Arabia

**Keywords:** AI-supported feedback, artificial intelligence in education, ChatGPT, handball skills, higher education, physical education, reciprocal teaching style, teaching

## Abstract

**Introduction:**

Digital technologies and artificial intelligence offer new opportunities to enhance teaching and learning processes in physical education. However, empirical evidence on the pedagogical use of ChatGPT-based feedback in skill-based sport instruction remains limited. This study examined the effectiveness of integrating ChatGPT-based digital feedback within the Reciprocal Teaching Style (RTS) to improve fundamental handball skills and students' attitudes in higher education.

**Methods:**

A randomized pre-test–post-test control group design was used. Fifty-six undergraduate students enrolled in a third-level university handball course, with no prior formal handball training, were randomly allocated to either an experimental group receiving ChatGPT-based digital feedback embedded within Reciprocal Teaching Style or a control group using reciprocal teaching with peer feedback only. Both groups completed a structured 15-session instructional program over one academic semester. ChatGPT was used to generate structured corrective feedback based on predefined performance criteria, with mandatory manual analysis and human supervision to verify feedback accuracy. Data were collected using standardized physical fitness tests, expert-validated performance checklists assessing seven fundamental handball skills, and a students' attitudes scale. Analysis of covariance was conducted while controlling for pre-test scores.

**Results:**

Statistically significant differences were found in favor of the experimental group across all assessed handball skills (*p* < .001), with medium to very large effect sizes (partial *η*^2^ = .33–.87). All results remained significant after Bonferroni adjustment, and *post-hoc* power analysis indicated high statistical power (≥.95). Students receiving ChatGPT-based feedback demonstrated higher mean scores across cognitive, affective, and behavioral attitude dimensions, including motivation, enjoyment, perceived usefulness, and intention to continue using AI-supported feedback.

**Conclusion:**

Integrating ChatGPT-based digital feedback within reciprocal teaching enhances technical skill acquisition and supports favorable learning-related attitudes in university physical education. This approach provides a practical pedagogical model for improving feedback quality and instructional efficiency in skill-based sport courses, particularly in large-class settings, when supported by structured procedures and human oversight.

## Introduction

Handball is a dynamic team sport in the university physical education curriculum. It requires agility, coordination, and tactical awareness. Effective performance depends on mastering core motor skills, including passing, catching, dribbling, shooting, faking, defensive actions, and goalkeeping. These skills are often difficult for novice learners to acquire (1, 2).

University beginners frequently lack prior exposure to the game. Instructional time is also limited, which restricts opportunities for technical development (3–5). For this reason, structured instructional approaches are essential. Such approaches should maximize practice time and provide immediate and meaningful feedback (6, 7).

Feedback is central to motor learning. It supports error detection and guides movement refinement (8, 9). In physical education contexts, the timing and accuracy of feedback influence the rate of skill acquisition and learners' confidence (3, 10). Empirical evidence further indicates that structured feedback contributes to both immediate performance gains and long-term retention (2, 11). This is particularly relevant for novice handball players, who must coordinate complex motor actions under dynamic conditions (12).

Among the variety of teaching approaches in PE, Mosston and Ashworth's Reciprocal Teaching Style (RTS) has been widely studied for its potential to foster peer-supported learning (13, 14). In this style, students alternate between the role of performer and observer, with observers using a structured criteria sheet to provide feedback on performance execution (15). This method empowers students to take responsibility for learning while simultaneously promoting communication, collaboration, and reflective practice (7). Evidence suggests that reciprocal teaching enhances motor skill learning in a variety of sports and contexts by ensuring that learners are consistently engaged in both action and observation (15).

The RTS has been associated with improvements in technical proficiency, autonomy, and critical thinking (14, 16). However, its effectiveness often depends on the quality and consistency of peer feedback. Observers with limited experience or uncertainty may provide feedback that lacks accuracy or pedagogical depth (16). Additionally, variability in peer-delivered feedback can undermine potential learning gains and reduce long-term efficacy. These limitations suggest a need for supplementary scaffolding mechanisms such as structured training or digital tools to support observers in delivering reliable guidance. Recent studies highlight that carefully scaffolded interventions, including models-based frameworks and guided practice, can significantly enhance the fidelity and impact of RTS instruction (17).

Technological innovations have been introduced to address instructional constraints in physical education. Artificial intelligence (AI) tools are increasingly used to support individualized feedback and performance monitoring, but their effectiveness depends on structured implementation and pedagogical alignment (18, 19). In sports education, motion analysis software and wearable technologies have shown value when integrated into guided practice settings (20, 21). For example, Kinovea has been validated as a biomechanical video analysis tool that enables learners to visualize movement execution and adjust technique under supervised conditions (22).

Generative AI models such as ChatGPT have recently been explored in educational contexts. Their capacity to generate context-sensitive responses may support instructional planning and feedback processes (23, 24). In PE and teacher preparation, emerging studies indicate that ChatGPT can assist with lesson design and the formulation of corrective cues when guided by clear instructional criteria (25–27). However, systematic reviews caution that effective use requires critical evaluation, clear institutional policies, and human oversight (28–30). Its accessibility may increase its practicality in higher education, particularly in contexts with limited instructional resources.

Recent research suggests that ChatGPT can provide feedback that aligns with several dimensions of human instructional support, including clarity, accuracy, and tone (31). However, these outcomes depend on the quality of prompts, task design, and appropriate supervision. Immediate and consistent feedback may enhance formative learning processes when integrated within structured instructional settings.

In the present study, ChatGPT was used primarily to provide structured knowledge of performance feedback (9). The feedback focused on qualitative aspects of movement execution, such as body positioning, timing, and coordination, rather than only reporting task outcomes. It included corrective cues aligned with predefined technical criteria within the RTS. The feedback was descriptive and corrective rather than merely prescriptive, and it was delivered immediately following performance analysis under teacher supervision.

Meta-analytic evidence indicates overall positive effects on academic performance, higher-order thinking, and cognitive load in higher education contexts (32). In physical education, preliminary findings suggest that ChatGPT may support participation and interactive learning when embedded within guided practice (33). In applied sport settings, it has been used to generate resistance training prescriptions and provide preliminary feedback. These applications show potential, but they require human oversight to ensure contextual accuracy and safety (34).

Students' perceptions of AI also warrant careful consideration. Large-scale and country-level surveys show that learners generally view AI tools positively for efficiency and accessibility, while still expressing concerns about academic integrity, over-reliance, and impacts on critical thinking (35, 36). In PE contexts, affective and motivational attitudes such as enjoyment, perceived usefulness, and autonomous forms of motivation are key determinants of sustained engagement and willingness to participate in physical activity (37, 38). Thus, any instructional innovation must be evaluated not only for technical outcomes but also for its influence on the affective dimensions of learning.

Empirical evidence on the integration of free-access generative AI tools into established teaching strategies in physical education remains limited (19). In particular, it is unclear whether ChatGPT can be systematically embedded within the Reciprocal Teaching Style to improve the consistency and accuracy of peer feedback in skill-based settings. There is also limited evidence regarding its influence on students' attitudes toward learning in practical sport environments (39). This gap limits evidence-based guidance for teachers and reduces understanding of how generative AI can function within skill-based pedagogical models.

In response, the present study seeks to evaluate the effectiveness of ChatGPT-based digital feedback within RTS in improving fundamental handball skills and students' attitudes among university beginners. By bridging established pedagogy with emerging technology, the study contributes to evidence-based innovation in sports education. Moreover, it provides timely insights into how generative AI can serve as a supportive tool for motor skill learning and affective development in higher education settings, advancing both theoretical knowledge and practical applications in the field. This study is also directly relevant to the educational practices in colleges and departments of sport sciences and physical activity in Saudi Arabia, where the integration of innovative technologies such as ChatGPT aligns with national efforts to enhance teaching quality, promote active learning, and prepare graduates who can contribute effectively to the goals of Saudi Vision 2030. Furthermore, it resonates with global priorities on sustainability in higher education by promoting adaptable, technology-driven practices that can support long-term improvements in teaching and learning. This study contributes to sustainable digital education by proposing a scalable, low-cost AI-supported feedback model that enhances teaching effectiveness and learner engagement in higher education.

## Objectives of the study

1. To evaluate the effectiveness of integrating ChatGPT-based digital feedback within RTS in improving fundamental handball skills among university beginners.
2. To investigate students' attitudes toward the use of ChatGPT-based digital feedback within RTS in physical education.

## Research questions

*RQ_1_*. Does ChatGPT-based digital feedback within RTS improve the acquisition of fundamental handball skills compared to traditional RTS with peer feedback?

*RQ_2_*. What are students' attitudes toward ChatGPT-based digital feedback within RTS?

## Research hypotheses

*H_1_*. Students who receive ChatGPT-based digital feedback within RTS will demonstrate significantly greater improvements in fundamental handball skills than those who receive only peer feedback.

*H_2_*. Students in the experimental group will report positive attitudes toward ChatGPT-based digital feedback within RTS.

**Figure 1:**
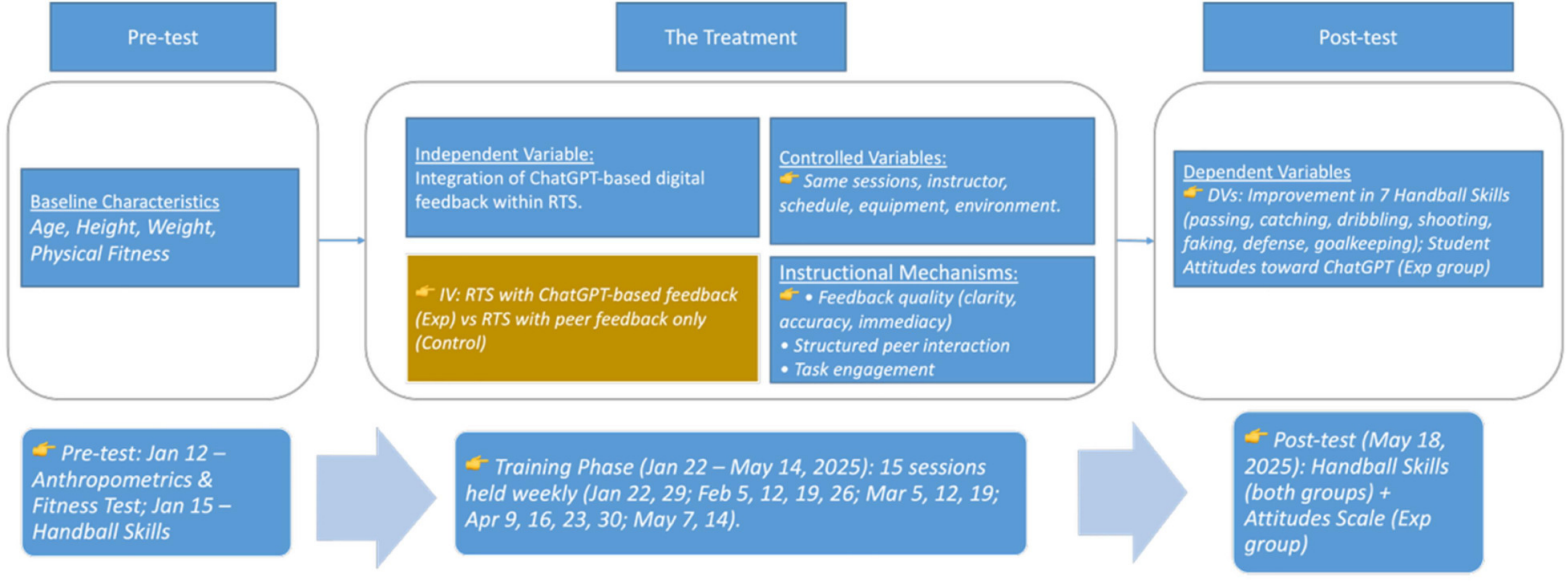
Conceptual framework of ChatGPT-based digital feedback within RTS and study timeline.

Figure 1 illustrates the pre test, treatment, and post test phases of the study. The independent variable was the integration of ChatGPT-based digital feedback within the Reciprocal Teaching Style. The dependent variables were improvement in fundamental handball skills and students' attitudes. The model includes instructional mechanisms related to feedback clarity, accuracy, and immediacy, as well as structured peer interaction and task engagement. These elements represent theoretical processes embedded in the instructional design and were not tested as statistical mediators. Standardized training conditions were maintained across groups. The same instructor, number of sessions, schedule, equipment, and learning environment were used. Baseline characteristics, including age, height, weight, and general physical fitness, were recorded prior to the intervention to confirm group equivalence. Baseline measurements were conducted on January 12 and 15, 2025. Fifteen instructional sessions were delivered between January 22 and May 14, 2025. Post testing was conducted on May 18, 2025.

## Theoretical framework

### Theoretical foundation

The present study is grounded in the OPTIMAL theory of motor learning (10). This theory integrates motivational and attentional mechanisms to explain how motor performance can be enhanced. It proposes that learning improves when three conditions are supported: autonomy, positive expectancies, and an external focus of attention. Empirical evidence has consistently shown that directing attention toward movement effects enhances performance and retention (40, 41). Similarly, providing learners with control over aspects of practice, such as feedback timing, strengthens learning efficiency (42, 43). Positive performance expectancies have also been linked to improved motor acquisition (44).

This framework is particularly relevant to the present study, as the RTS involves shared responsibility and structured feedback, while the integration of digital feedback introduces an additional source of guidance. Together, these elements align conceptually with the conditions outlined in the OPTIMAL theory and provide a theoretical basis for examining both performance outcomes and learner attitudes.

### Motor learning and feedback in PE

Feedback is essential in motor skill learning. It allows learners to compare current performance with the intended goal (8). Empirical evidence confirms its effectiveness. Zhou et al. (2) reported that students who received feedback outperformed those who did not. Han et al. (3) found moderate effect sizes, particularly when feedback was corrective and visually supported.

The timing and format of feedback influence learning outcomes. Video-based feedback has shown stronger effects than verbal-only feedback in complex tasks (45). Self-controlled feedback also enhances learning efficiency (42, 43).

The distinction between knowledge of results (KR) and knowledge of performance (KP) remains central. Knowledge of performance provides information about movement execution. It is especially important for novices. Knowledge of results provides outcome-based information. Sharma et al. (46) confirmed that both forms support motor learning. Stronger effects were observed when performance-based feedback was combined with visual input.

Technological tools have expanded feedback delivery. Multimodal systems increase awareness of movement characteristics (47). Kinovea has demonstrated reliability in movement analysis (22). Collectively, this evidence indicates that effective feedback should be structured, timely, and performance-oriented. This principle supports the exploration of digitally assisted feedback in the present study.

### RTS in PE

The RTS is part of Mosston and Ashworth's Spectrum of Teaching Styles (48). Students alternate between performer and observer roles. Observers use criteria sheets to guide feedback. This structure promotes shared responsibility.

Research supports its effectiveness. Byra and Marks (49) found improvements in feedback accuracy. Chatoupis (50) reported superior performance outcomes compared with the practice style. Theodosiou et al. (51) observed greater enjoyment and effort. Alhayja et al. (52) reported improvements in physical fitness outcomes. Reviews emphasize the value of student-centered instruction in physical education (53, 54).

However, the effectiveness of RTS depends on feedback quality. When observers lack experience, feedback accuracy may decline. This limitation creates the need for structured support mechanisms.

### AI and ChatGPT in PE

AI tools have been introduced into PE to support movement monitoring and feedback. Reviews highlight their instructional potential (55). Real-time pose detection systems have improved movement fluency and engagement Ma et al. (6).

Generative models such as ChatGPT have been applied in educational contexts. Genç (56) reported its use in designing training programs and generating instructional materials. Zhang and Liu (33) observed improvements in participation and psychological outcomes. However, comparative research shows that AI feedback does not consistently match human expertise in nuanced performance evaluation (57, 58). Trust in AI feedback depends on transparency and supervision (59).

In the present study, ChatGPT is conceptualized as providing structured KP feedback. It generates corrective cues related to movement execution. It reinforces task criteria. It maintains a supportive tone. Its role is to stabilize feedback consistency within the Reciprocal Teaching Style.

### Research Gap and rationale

Handball requires coordinated execution of fundamental skills under dynamic conditions. Structured practice and reciprocal teaching approaches improve these skills (60–62). However, peer-delivered feedback may lack consistency when learners are inexperienced.

Empirical evidence examining the integration of generative AI within established pedagogical models in physical education remains limited. To our knowledge, very limited experimental research has investigated whether ChatGPT can be embedded within the Reciprocal Teaching Style to enhance feedback quality in handball instruction. This gap limits evidence-based guidance for teachers and reduces understanding of how generative AI can function within structured motor learning environments.

The present study examines whether ChatGPT-supported knowledge-of-performance feedback within RTS improves fundamental handball skills and influences student attitudes in university physical education.

## Methodology

### Research design

This study used a randomized controlled pre test post test design. Participants were individually assigned to either an experimental group that received digital feedback within the Reciprocal Teaching Style or a control group that received reciprocal teaching with traditional peer feedback. Both groups completed identical pre test and post test assessments (63, 64).

### Participants

The sample consisted of 56 students enrolled in the third level of the Bachelor of Sport and Physical Activity Sciences program at Qassim University during the second semester of the 2024–2025 academic year. All participants were registered in the course SPOR241-Principles of Handball and its Applications, which includes a practical component on fundamental handball skills. Students had no prior structured training in handball beyond the course. Those with current injuries or who missed more than two sessions were excluded. Following eligibility screening, participants were individually randomized into two groups (28 control, 28 experimental) using a simple random allocation procedure in IBM SPSS Statistics. Student identification numbers were entered into the random number generator to ensure equal probability of assignment and minimize selection bias.

### Sample size justification

An *a priori* sample size justification was performed using G*Power 3.1 (65) for ANCOVA with two groups and one covariate. A large effect was assumed (*f* = 0.40), consistent with conventional benchmarks (66) and evidence from structured feedback research in motor learning and PE (2, 3). With *α* = .05 and *power* = .80, the required total sample was 52 participants. The present study included 56 students (28 per group), meeting this criterion.

### Ethical considerations

The study adhered to established ethical standards for research involving human participants. Approval was obtained from the Research Ethics Committee of the Department of Physical Education and Kinesiology, College of Education, Qassim University (Approval No. 01-462-12-01-2025). Prior to data collection, all students were provided with a written informed consent form that clearly explained the objectives, significance, and procedures of the study. The form emphasized the voluntary nature of participation, the confidentiality of responses, and the right to withdraw from the study at any time without penalty. The final section of the consent form included a declaration where students could explicitly indicate their agreement or refusal to participate. All data were collected, stored, and analyzed anonymously to ensure participant privacy and confidentiality.

### Instruments and measures

1. General Physical Fitness Test Battery: To verify group equivalence and homogeneity, all participants completed the standardized admission fitness test battery adopted and approved by the program administration. This battery is officially validated and enjoys high psychometric quality. Content validity was confirmed through discrimination validity testing, with a differentiation index of 0.72, while reliability was established through the split-half method, yielding a coefficient of 0.88 (67, 68).
  a) Speed: 50 m sprint from a crouch start.
  b) Muscular endurance: Push-ups in 30 s.
  c) Explosive power: Standing broad jump.
  d) Agility: Zigzag run between cones.
  e) Flexibility: Sit-and-reach test.
  f) Coordination: Jumping between numbered circles.
2. Fundamental Handball Skills Performance Checklists: Researcher-developed checklists were used to assess the main fundamental handball skills included in the instructional program, namely passing, catching, dribbling, shooting, faking, defensive skills, and goalkeeping. Each skill was evaluated on a five-point scale (1 = very poor to 5 = excellent). All performances were video-recorded. The recordings were coded and anonymized prior to evaluation to conceal group membership. Three expert referees independently evaluated the videos. All referees held doctoral degrees in physical education with specialization in handball and had more than ten years of experience in teaching and coaching at the university level. They were blinded to the study hypotheses and group allocation. To establish content validity, the checklists were reviewed by specialists in handball and physical education. For reliability, the instruments were piloted with a separate exploratory sample of students who had completed the course in the previous semester and were not part of the main study. Inter-rater reliability coefficients exceeded.85, confirming consistency in scoring (69, 70). Final scores were calculated as the mean of the three evaluators' ratings.
3. Students' Attitudes Scale: The Students' Attitudes Scale was developed to assess students' perceptions of ChatGPT-based digital feedback within the Reciprocal Teaching Style. The scale included 20 items distributed across three domains: cognitive, affective, and behavioral. The three domains were selected based on established models of attitude structure that conceptualize attitudes as cognitive, affective, and behavioral components and link them to the attitude behavior relationship in educational settings (71, 72). Content validity was established through expert review by specialists in handball and physical education. The experts evaluated item clarity, relevance, and alignment with the study objectives. Minor wording revisions were made prior to pilot testing. The scale was piloted with 48 students from a previous academic cohort who were not included in the main study. The independent variable was applied for two weeks under conditions comparable to those of the main experiment, after which the scale was administered. Internal consistency validity was examined using item-total correlation coefficients. Correlation values ranged from.31 to.63 and were statistically significant at *p* < .001. Reliability was assessed using Cronbach's alpha (69, 70). The overall alpha coefficient was.87, and all domain-level alpha values exceeded.70. Detailed item-total correlations and alpha if item deleted statistics are presented in Supplementary Material S1, while domain reliability coefficients are reported in Supplementary Material S2. These results support the use of the scale in the main study (68).

### Procedures

1. Pre-test (Baseline Assessment):
  The baseline assessment was conducted on January 12 and 15, 2025. It included three components:
  − *Anthropometric measurements* (height and weight) recorded to describe the sample and verify group equivalence.
  − *General Physical Fitness Composite Score:* Raw scores from the six admission fitness tests (speed, muscular endurance, explosive power, agility, flexibility, coordination) were standardized (z-scores), rescaled to a 0–10 band for each test, and summed to form a 0–60 composite score (higher = better). The composite demonstrated strong psychometric quality and was used both to verify baseline equivalence and as a covariate in outcome analyses.
2. *Fundamental handball skills assessment:* Researcher-developed checklists were used to assess the seven target skills (passing, catching, dribbling, shooting, faking, defensive skills, and goalkeeping). Assessments were video-recorded and scored independently by three expert referees to ensure objectivity and reliability. General Physical Fitness Test Intervention (Training Phase):
  The instructional program was implemented across 15 sessions between January 22 and May 14, 2025**,** following the official SPOR241 schedule. Both groups practiced under identical conditions with the same instructor (Associate Professor of Physical Education).
  − *Experimental group (n* *=* *28):* Received the RTS supported by ChatGPT-based digital feedback.
  − *Control group (n* *=* *28):* Received the RTS with traditional peer feedback only.
3. Educational Program:
  The program progressively developed handball skills in six instructional blocks:
  − *Weeks 1–3 (Jan 22—Feb 12):* Ball familiarization and manipulation; introduction to catching and passing.
  − *Weeks 4–6 (Feb 19—Mar 5):* Catching and passing (chest, bounce, overhead).
  − *Weeks 7–9 (Mar 12—Apr 2):* Dribbling (straight line, zigzag, shielded) with continuation of catching and passing.
  − *Weeks 10–11 (Apr 9—Apr 16):* Shooting (overarm, lob, bounce) and faking (passing and shooting fakes).
  − *Weeks 12–13 (Apr 23—Apr 30):* Advanced shooting, body fakes, defensive basics, and goalkeeping.
  − *Weeks 14–15 (May 7—May 14):* Zone and switch defense, advanced goalkeeping, and small-sided games.
  A condensed overview is presented in Table 1, while the full instructional units including objectives, drills, and observation sheets are available in the Supplementary Information (Appendix S1).
Post-test (Outcome Assessment):
  Conducted on May 18, 2025. The same fitness test battery and performance checklists were administered. In addition, the Students' Attitudes Scale was applied to the experimental group only.

### AI-Supported digital feedback protocol within the reciprocal teaching style

1. *Technological Platform Specification:* The intervention utilized the official ChatGPT mobile application (free-access public version) powered by GPT-4o (OpenAI, May 2024 release). Students accessed the system through their personal smartphones (Android and iOS devices) using campus Wi-Fi. No paid subscription tiers (e.g., Plus or Pro), plugins, API integrations, or third-party analytical tools were employed. The default publicly available model active during the Spring 2025 semester was used, and students did not manually select model versions. ChatGPT was used exclusively for structured corrective feedback generation based on observer-written error descriptions.
2. *General Session Structure:* Each session lasted 90 min and followed a consistent structure: approximately 10 min warm-up, 10 min physical preparation, 50 min main skill development, 15 min application/game phase, and 5 min cool-down. AI-supported feedback was integrated exclusively within the main skill development phase. No AI interaction occurred during warm-up, physical preparation, application activities, or cool-down. The first instructional session was dedicated to orientation and procedural training.
3. *Instructional Framework:* The intervention was implemented within the Reciprocal Teaching Style. Following the instructor-led explanation and demonstration at the beginning of the main skill development phase, students worked in fixed dyads consisting of a performer and an observer. The performer executed the assigned skill, while the observer evaluated performance using a structured checklist aligned with the unit's technical criteria. Based on the observed performance, the observer provided corrective feedback supported by AI-generated suggestions, after completing the mandatory manual analysis procedures. Roles were exchanged systematically after each reciprocal practice cycle. The instructor maintained full instructional authority throughout the session, including verification of technical accuracy, supervision of reciprocal procedures, and enforcement of safety and pedagogical guidelines.
4. *Mandatory Manual Analysis Prior to AI Interaction:* Before interacting with ChatGPT, observers were required to re-observe the performed skill at least once, complete the structured observation checklist, identify the specific unmet performance criterion, and write a concise error description (1–2 sentences).‏ AI interaction was strictly prohibited prior to completing manual analysis to preserve evaluative responsibility and cognitive engagement.
5. *Standardized Prompt Template:* A fixed printed prompt template was distributed to all students and used consistently throughout the semester. Students were instructed not to modify the structure. Only the “Observed error” section was student-generated. The exact prompt used in the study was:
  You are a physical education instructor.
  Skill: [Skill Name]
  Correct Technical Criteria:
    − [Criterion 1]
    − [Criterion 2]
    − [Criterion 3]
    − [Criterion 4]
  Observed error:
  [Student-written description]
  Required:
    − Identify the error precisely.
    − Explain its mechanical effect on performance.
    − Provide a corrective instruction.
    − Suggest one simple remedial drill.
    − Respond concisely and professionally.
    Observers were limited to one AI query per evaluation cycle to prevent over-reliance and ensure procedural consistency.
6. *Time Allocation Within the Skill Phase:* The main skill development phase lasted 50 min and was fully implemented using the Reciprocal Teaching Style. The first 10 min were led by the instructor and dedicated to skill explanation, demonstration of correct technical criteria, clarification of observation checklist items, and reinforcement of reciprocal teaching procedures and safety instructions. The remaining 40 min were organized into repeated reciprocal practice cycles. Each cycle included a short period of drill execution and repetition, followed immediately by reciprocal evaluation and AI-supported corrective feedback, then brief role exchange. Across the session, approximately 30 min were devoted to drill execution and repetition, 15–18 min to reciprocal evaluation cycles including AI-supported feedback, and 2–3 min to role exchange transitions. The average AI interaction time per cycle was approximately 2–3 min. The AI-supported component did not extend total session duration.
7. *Human Validation Layer:* AI-generated feedback was validated through three mechanisms. First, the observer reviewed and edited the AI output to ensure alignment with checklist criteria. Second, the instructor audited AI-supported feedback cases per session to verify accuracy of error identification and alignment with technical performance standards. Third, the performer re-executed the skill after receiving feedback, and observable technical adjustment served as functional validation. AI outputs did not determine performance grading.
8. *AI Usage Constraints and Dependency Control:* AI use was restricted to structured corrective feedback generation only. Manual checklist completion was mandatory before AI interaction. Only written descriptions were entered into the system, and no multimedia files were uploaded. Verbatim submission of AI output without observer revision was discouraged. AI-generated responses were not used for scoring decisions. Free-tier message limits did not interfere with implementation due to controlled query frequency.
9. *Procedural Consistency and Model Stability:* Although OpenAI may update background model performance during a semester, the following procedural elements remained constant: standardized prompt template, structured observation checklist, reciprocal role exchange structure, instructor supervision protocol, session duration, and free mobile access mode. These controls ensured procedural integrity and full replicability of the intervention.
10. *Ethical Safeguards:* No personal identifiers were entered into ChatGPT. AI interaction logs were not archived. Participation consent was obtained from students. Instructor supervision ensured safety during physical contact drills.

### Data analysis

Data were analyzed using IBM SPSS Statistics version 25 (73). Independent samples t-tests tested baseline equivalence. ANCOVA was applied for post-test comparisons using pre-test scores as covariates. Descriptive statistics and independent samples t-tests analyzed student attitudes. Statistical significance was set at *p* < .05.

**Table 1:** Summary of the 15-session instructional program (SPOR241).

Weeks	Focus skills	Examples of Activities
1–3	Ball familiarization and manipulation; intro to catching & passing	Dribbling drills; basic catching; chest pass
4–6	Catching and passing (chest, bounce, overhead)	Partner catching; bounce pass; overhead pass
7–9	Dribbling with continuation of catching and passing	Dribbling under pressure; catching in pairs
10–11	Shooting and faking	Overarm shot; lob shot; fake and dribble
12–13	Advanced shooting, fakes, defense, goalkeeping	Rotation shot; body fake; 1v1 defense; reaction saves
14–15	Advanced defense and small-sided games	Zone defense; diving saves; 3v3 and 5v5 games

## Results

### Research question 1/hypothesis 1

*RQ_1_.* Does ChatGPT-based digital feedback within RTS improve the acquisition of fundamental handball skills compared to traditional RTS with peer feedback?

*H_1_.* Students who receive ChatGPT-based digital feedback within RTS will demonstrate significantly greater improvements in fundamental handball skills than those who receive only peer feedback.

#### Pre-Test equivalence

Independent-samples *t*-tests were conducted to compare the control and experimental groups on anthropometric measures, physical fitness, and fundamental handball skills prior to the intervention. No statistically significant differences were found (*p* > .05), confirming baseline equivalence and homogeneity between groups (Table 2).

**Table 2:** Pre-test descriptive statistics and independent samples *T*-test.

Variable	Group	*N*	*M*	*SD*	Min	Max	*t*	*p*
Height (cm)	Exp	28	178	3.3	171	183	0.12	0.906
	Con	28	177.9	3.9	171	183		
Weight (kg)	Exp	28	74.2	4	68	82	0.07	0.946
	Con	28	74.3	4.7	68	83		
Fitness Composite	Exp	28	40.4	4.2	32	47	−0.02	0.984
	Con	28	40.4	4.1	32	47		
Passing	Exp	28	2.2	0.13	1.9	2.3	0.56	0.579
	Con	28	2.18	0.12	1.8	2.3		
Catching	Exp	28	2.17	0.14	1.9	2.3	−0.48	0.633
	Con	28	2.19	0.13	1.8	2.3		
Dribbling	Exp	28	2.21	0.15	1.9	2.3	0.27	0.789
	Con	28	2.2	0.13	1.8	2.3		
Shooting	Exp	28	2.19	0.14	1.9	2.3	0.21	0.833
	Con	28	2.18	0.15	1.8	2.3		
Faking	Exp	28	2.16	0.15	1.8	2.3	−0.18	0.857
	Con	28	2.17	0.14	1.8	2.3		
Defense	Exp	28	2.18	0.13	1.9	2.3	−0.39	0.699
	Con	28	2.2	0.12	1.8	2.3		
Goalkeeping	Exp	28	2.2	0.14	1.9	2.3	0.24	0.812
	Con	28	2.19	0.13	1.8	2.3		
Skills Total Avg	Exp	28	2.19	0.12	1.9	2.3	0	0.997
	Con	28	2.19	0.11	1.8	2.3		

Exp, Experimental group; Con, Control group.

#### Post-Test results and ANCOVA

Table 3 displays the post-test descriptive statistics (means and standard deviations) for both groups alongside ANCOVA results with pre-test scores as covariates. The experimental group consistently achieved higher scores than the control group across all measured skills. ANCOVA confirmed statistically significant differences favoring the experimental group (*p* < .001). Effect sizes (Partial *η²*) ranged from medium to very large, with the composite score demonstrating the strongest effect (*η*^2^ = .873).

**Table 3:** Post-Test descriptive statistics and ANCOVA results for handball skills.

Skills	Control (M ± SD)	Experimental (M ± SD)	Adjusted Diff (95% CI)	*F*(1,53)	*p*	Partial *η*^2^	MSE
Passing	2.36 ± 0.18	2.92 ± 0.30	0.546 [0.406, 0.686]	60.80	<.001	0.534	0.063
Catching	2.29 ± 0.19	2.89 ± 0.36	0.594 [0.438, 0.749]	58.63	<.001	0.525	0.084
Dribbling	2.35 ± 0.20	2.91 ± 0.29	0.562 [0.427, 0.697]	69.77	<.001	0.568	0.063
Shooting	2.39 ± 0.20	2.85 ± 0.33	0.475 [0.325, 0.625]	40.43	<.001	0.433	0.076
Faking	2.42 ± 0.20	2.76 ± 0.29	0.344 [0.210, 0.478]	26.61	<.001	0.334	0.063
Defense	2.37 ± 0.18	2.87 ± 0.26	0.502 [0.384, 0.619]	73.56	<.001	0.581	0.050
Goalkeeping	2.35 ± 0.13	2.77 ± 0.39	0.412 [0.251, 0.572]	26.47	<.001	0.333	0.085
Composite	2.36 ± 0.07	2.85 ± 0.12	0.491 [0.440, 0.543]	363.25	<.001	0.873	0.009

ANCOVA covariate, Pre-test scores.

The ANCOVA results indicated statistically significant differences between the experimental and control groups across all fundamental handball skills after controlling for pre-test scores. All effects remained significant at *p* < .001 and under the Bonferroni-adjusted criterion (*α* = .007). Large effect sizes were observed for Defense ($\eta_{p}^{2}=.581$), Dribbling ($\eta_{p}^{2}=.568$), and Passing ($\eta_{p}^{2}=.534$). Catching ($\eta_{p}^{2}=.525$) and Shooting ($\eta_{p}^{2}=.433$) also demonstrated substantial effects, while Faking ($\eta_{p}^{2}=.334$) and Goalkeeping ($\eta_{p}^{2}=.333$) showed moderate-to-large effects. The adjusted mean differences consistently favored the experimental group, and all 95% confidence intervals excluded zero. The composite score yielded a very large overall effect size (*F*(1,53) = 363.25, $\eta_{p}^{2}=.873$), indicating a strong cumulative improvement across skills (63, 74).

To further support the adequacy of these findings, a *post-hoc* power analysis was conducted using G*Power 3.1 (65, 75). Based on the observed effect sizes, the statistical power (*1—β*) exceeded.95 for all skills and reached 1.00 for most outcomes, including the composite score. According to Cohen (66), this demonstrates that the study was sufficiently powered to detect medium-to-large effects, ensuring the reliability and robustness of the results.

These findings support the study's first hypothesis (*H_1_*), providing strong evidence that digital feedback via ChatGPT enhances skill acquisition beyond traditional peer feedback within the Reciprocal Teaching framework.

### Research question 2/hypothesis 2

*RQ_2_*. What are students' attitudes toward ChatGPT-based digital feedback within RTS?

*H_2_.* Students in the experimental group will report positive attitudes toward ChatGPT-based digital feedback within RTS.

To assess students' perceptions toward the integration of ChatGPT-based digital feedback within RTS, a 20-item attitudes scale was administered to the experimental group (*n* = 28). The scale included three main axes cognitive, affective, and behavioral further divided into sub-dimensions such as effectiveness, usefulness, enjoyment, motivation, ease of use, autonomy, collaboration, trust, and willingness to continue. Responses were rated on a 5-point Likert scale (1 = strongly disagree to 5 = strongly agree). The scale was implemented using a Google Form immediately after the final instructional session, ensuring that students' responses reflected their direct and recent experiences with the intervention. Descriptive statistics (means, standard deviations, and interpretive categories) were calculated for each item, while Cronbach's alpha coefficients were used to examine the internal consistency of the sub-dimensions and the overall scale. The results are presented in Table 4.

**Table 4:** Descriptive statistics and reliability of students' attitudes toward ChatGPT-based digital feedback within RTS (*n* = 28).

Axis/Sub-dimension	Item Statement	*M*	*SD*	Interpretation
Cognitive—Effectiveness (*α* = .78)	ChatGPT simplified the understanding of handball skills.	4.1	0.7	High
	ChatGPT improved retention and comprehension.	4	0.8	High
	ChatGPT provided feedback that helped correct mistakes.	3.9	0.7	High
Cognitive—Usefulness (*α* = .72)	ChatGPT made the lessons more meaningful.	3.8	0.8	High
	ChatGPT feedback supported the achievement of objectives.	3.9	0.6	High
Affective—Enjoyment (*α* = .75)	Using ChatGPT during lessons was enjoyable.	4.2	0.6	Very High
	ChatGPT enriched my learning experience.	4.1	0.7	High
Affective—Motivation (*α* = .80)	ChatGPT feedback increased my motivation to learn.	4.3	0.7	Very High
	ChatGPT encouraged me to participate actively.	4.2	0.7	Very High
	ChatGPT feedback increased my confidence.	4.1	0.8	High
Behavioral—Ease of Use (*α* = .76)	ChatGPT was easy to use and quick to access.	4.1	0.6	High
	ChatGPT feedback was clear and immediate.	4	0.7	High
Behavioral—Autonomy (*α* = .74)	ChatGPT helped me learn skills independently.	3.9	0.7	High
	ChatGPT encouraged me to review outside class.	3.8	0.8	High
Behavioral—Collaboration (*α* = .77)	ChatGPT supported peer-to-peer interaction.	4	0.6	High
	ChatGPT improved peer observations.	4.1	0.7	High
Behavioral—Trust & Reliability (*α* = .71)	I trust the accuracy of ChatGPT's feedback.	3.9	0.7	High
	ChatGPT feedback was consistent and reliable.	3.8	0.8	High
Behavioral—Willingness (*α* = .79)	I would like to continue using ChatGPT.	4.4	0.6	Very High
	I would recommend ChatGPT to others.	4.3	0.6	Very High
Total Scale (20 items, *α* = .91)	Overall attitudes toward ChatGPT	3.9	0.6	High

Participants in the experimental group (*n* = 28) expressed overall positive attitudes toward ChatGPT-based digital feedback within RTS. The total mean score was 77.5 (*SD* = 6.2) out of a possible 100, indicating a high level of acceptance. Reliability analysis showed excellent internal consistency for the overall scale (Cronbach's *α* = .91) and acceptable to good levels for all sub-dimensions (*α* = .71–.80).

At the axis level, the Behavioral dimension achieved the highest mean (*M* = 38.8, *SD* = 3.6), reflecting strong agreement regarding ease of use, autonomy, collaboration, trust, and willingness to continue. Within this dimension, the highest-rated items were “*I would like to continue using ChatGPT”* (*M* = 4.4, *SD* = 0.6) and “*I would recommend ChatGPT to others”*(M = 4.3, *SD* = 0.6), both classified as “very high.”.

The Affective dimension (*M* = 19.6, *SD* = 2.3) also received highly positive ratings. Students particularly valued the motivational aspects, with items such as “*ChatGPT feedback increased my motivation to learn”* (*M* = 4.3, *SD* = 0.7) and “*ChatGPT encouraged me to participate actively”* (*M* = 4.2, *SD* = 0.7). Enjoyment-related items also reached high to very high levels, suggesting that ChatGPT enhanced engagement and the affective quality of lessons.

The Cognitive dimension (*M* = 19.1, *SD* = 2.1) was positively evaluated, with students reporting that ChatGPT simplified skill learning and improved comprehension. Effectiveness and usefulness sub-dimensions both scored in the “high” range, indicating that learners perceived ChatGPT as a supportive tool for understanding and applying handball skills.

Overall, these results demonstrate that integrating ChatGPT into RTS not only facilitated skill learning but also enhanced motivation, enjoyment, and willingness to continue using AI-assisted.

## Narrative summary

The findings strongly support both hypotheses of the study. ChatGPT-based digital feedback within RTS significantly enhanced the acquisition of fundamental handball skills, as evidenced by higher post-test scores and medium-to-very large effect sizes across all measured skills. Students in the experimental group not only demonstrated superior technical performance but also reported highly positive attitudes toward the integration of ChatGPT, particularly in terms of clarity, usefulness, motivation, and willingness to continue. These outcomes highlight that embedding generative AI into RTS can effectively overcome the limitations of inconsistent peer feedback, thereby ensuring more accurate, immediate, and structured guidance. Beyond confirming the theoretical value of combining AI tools with established pedagogical strategies, the results underscore important practical implications: integrating ChatGPT into physical education programs can enrich students' learning experiences, foster engagement and collaboration, and ultimately strengthen the effectiveness of skill-based instruction in higher education contexts. In line with Saudi Vision 2030, these results further demonstrate how innovative educational technologies can contribute to improving the quality of university-level physical education and developing future-ready graduates. They also highlight the potential for advancing teaching strategies in sport teacher preparation programs, ensuring that future educators are equipped with effective, technology-enhanced pedagogical practices. Looking ahead, these findings pave the way for further exploration of AI integration in sport science curricula, opening new opportunities to innovate teaching and learning in university-level physical education.

## Discussion

This section discusses the findings in relation to the two research questions: first, the impact of ChatGPT-based digital feedback on the acquisition of fundamental handball skills (RQ1), and second, students' attitudes toward this integration (RQ2).

The results showed that integrating ChatGPT-based digital feedback within the Reciprocal Teaching Style improved performance across all fundamental handball skills. Effect sizes ranged from medium to very large. The largest gains appeared in defense, dribbling, and passing, which require rapid adjustment and continuous decision-making. These findings indicate a meaningful instructional effect. However, the magnitude of improvement may also reflect contextual influences. The introduction of a new feedback tool may have increased students' attention and engagement. Peer interaction may have strengthened motivation and accountability during practice. These interpretations are consistent with evidence that structured feedback enhances learning in complex motor tasks (2).

The current findings are also consistent with previous research on the effectiveness of reciprocal teaching in handball. For example, Mustafa (60) and Elhachemi (62) both reported significant improvements in shooting and other fundamental skills through RTS-based interventions, confirming that structured peer observation supports technical development. However, the present study extends these findings by integrating a digital feedback component through ChatGPT. This hybrid model provided immediate, consistent, and structured guidance that complemented peer feedback, an element not addressed in the aforementioned studies. Similarly, Sahli et al. (61) emphasized the motivational benefits of peer encouragement during small-sided games, and our results suggest that ChatGPT-based feedback may play a comparable role by fostering student engagement and confidence.

In addition, students' positive attitudes toward ChatGPT can be further explained through theoretical frameworks of technology acceptance and motivation. According to the Technology Acceptance Model (TAM), perceptions of usefulness and ease of use are critical determinants of adoption (76); in this study, both dimensions were rated highly, reflecting students' belief that ChatGPT simplified learning and was accessible.

ChatGPT feedback may also be interpreted through the lens of Self-Determination Theory (SDT) (77). The digital feedback format allowed students to access guidance independently, which may have supported perceptions of autonomy. The corrective cues may have strengthened feelings of competence. The structured peer interaction within RTS may also have reinforced relatedness. These interpretations remain theoretical, as autonomy, competence, and relatedness were not directly measured. However, they provide a coherent framework for understanding why students reported high motivation and willingness to continue using the tool.

These findings are also consistent with evidence from systematic reviews and meta-analyses. Zhou et al. (2), in a systematic review of feedback in physical education, concluded that structured and consistent feedback yields moderate-to-large effects on motor skill learning, particularly for complex skills requiring rapid decision-making. Similarly, Deng et al. (32) conducted a meta-analysis of experimental studies using ChatGPT and reported overall positive effects on academic performance, higher-order thinking, and reduced cognitive load in higher education contexts. The present study aligns with these results by demonstrating medium-to-very large effect sizes in fundamental handball skills and by confirming the motivational and affective benefits of ChatGPT integration. Taken together, the current findings extend the generalizability of prior meta-analytic evidence by showing that generative AI can enhance both technical and affective outcomes in a skill-based, practice-oriented domain such as physical education.

It is noteworthy that all results remained statistically significant even after applying the Bonferroni correction (*α* = .007), which controls for inflated Type I error rates when conducting multiple comparisons (63, 74). This strengthens the robustness of the findings and confirms that the observed improvements are unlikely to be attributed to chance.

Furthermore, the *post-hoc* power analysis indicated statistical power values exceeding.95 for all skills and reaching 1.00 for the composite score, based on the observed effect sizes (65, 75). According to Cohen (66) criteria, these values demonstrate that the study was adequately powered to detect medium-to-large effects, thereby reinforcing the reliability and validity of the results.

Although the intervention produced large effect sizes, the magnitude of these improvements warrants careful interpretation. The introduction of a novel digital feedback tool may have increased attentional engagement during the early stages of the intervention. Increased engagement may have led to greater time on task, a well-established determinant of motor learning (9). The use of video-supported feedback may also have heightened performance awareness. The immediacy and structured nature of the feedback may have strengthened error detection and correction processes (8). These considerations suggest that the large composite effect likely reflects the interaction of instructional design and contextual learning conditions.

The outcomes can be interpreted through the OPTIMAL theory of motor learning (10). The integration of ChatGPT promoted learners' sense of autonomy, as they alternated between performer and observer roles while receiving personalized digital feedback. The immediacy and clarity of the feedback enhanced learners' expectancies for success, which in turn increased motivation and confidence. Furthermore, ChatGPT's corrective cues frequently highlighted external performance indicators (e.g., ball trajectory, release timing), directing learners' attention outward rather than inward toward body mechanics, thereby fostering more efficient and automatic learning processes (9).

The findings are also aligned with the Spectrum of Teaching Styles by (13, 48), where the reciprocal style is a central category. This style positions students as both performers and observers, fostering shared responsibility and collaborative learning (14, 15). However, one of its limitations is the variability of peer-delivered feedback, particularly when observers are novices (16). By embedding ChatGPT into RTS, the current study addressed this limitation, providing accurate, consistent, and immediate feedback while preserving the collaborative ethos of the spectrum. This demonstrates how digital tools can extend the application of Mosston's framework, producing a hybrid model that combines human interaction with AI-driven precision.

Beyond these primary frameworks, the findings are also consistent with constructivist and social learning perspectives, as students actively constructed knowledge through practice, observation, and feedback-supported modeling (3, 23).

From a practical standpoint, the integration of ChatGPT offers a scalable solution for universities facing challenges such as large class sizes and limited instructional time. Being free and easily accessible, ChatGPT provides an efficient resource for higher education settings, particularly in resource-constrained environments. This aligns with the global movement toward sustainable education (78, 79) and supports the development of blended learning models that combine traditional instruction with digital augmentation (80).

Regarding attitudes (RQ2), the results indicated that students expressed very high levels of motivation, enjoyment, and willingness to continue using ChatGPT. The highest-rated items were “I would like to continue using ChatGPT” and “I would recommend ChatGPT to others,” demonstrating strong acceptance. These findings align with (36), who reported that students worldwide generally perceive AI tools positively for their immediacy, accessibility, and motivational impact. Similarly, Zhang et al. (59) found that students considered hybrid human-AI feedback both objective and useful. In line with these results, Jud and Thalmann (80) confirmed that AI-enhanced coaching systems provide consistent and personalized support, further validating the current findings.

While the results are encouraging, some limitations must be acknowledged. Over-reliance on AI feedback may reduce the development of students' critical thinking skills if not accompanied by teacher guidance (30). Moreover, ChatGPT cannot fully replace human expertise in identifying subtle errors or addressing contextual nuances (58). Therefore, AI should be seen as a complement rather than a substitute for human instruction. Nonetheless, a major strength of this study lies in providing a practical model that addresses the key limitation of RTS peer feedback inconsistency while enhancing both cognitive and affective outcomes.

The findings open several avenues for future research. Further studies should investigate the long-term retention of motor skills learned with AI-supported feedback, compare ChatGPT with other AI-driven or multimodal feedback systems (e.g., video-based or motion-analysis tools) (81), and extend the application to other sports such as basketball or volleyball. Moreover, future research could explore ChatGPT's role in enhancing tactical decision-making and not only technical execution.

These outcomes align with the goals of Saudi Vision 2030, which emphasizes digital innovation, pedagogical modernization, and the preparation of future-ready graduates in physical education and sport sciences. The findings demonstrate how AI-supported feedback can contribute to instructional efficiency and educational transformation in university settings. Recent research further highlights the role of AI in improving training efficiency, supporting injury prevention, and enhancing sports analytics and decision-making (82–84).

In conclusion, integrating ChatGPT into RTS represents a powerful pedagogical innovation that bridges traditional and digital teaching practices. Grounded in both the OPTIMAL theory of motor learning and the Spectrum of Teaching Styles, this hybrid model enhances skill acquisition by supporting autonomy, positive expectancies, and external attentional focus, while also strengthening collaborative learning and shared responsibility. The positive attitudes reported by students confirm its motivational value and sustainability. Future studies should further examine long-term effects and explore multimodal AI tools to enrich physical education pedagogy.

## Conclusion

This study examined the integration of ChatGPT-based digital feedback within the RTS in university physical education. The findings indicate improvements in fundamental handball skills among students who received structured digital feedback. Students also reported positive attitudes and a willingness to continue using the tool. These results suggest that structured digital guidance can enhance the consistency of peer-supported instruction while supporting learner engagement. The model offers a practical approach for physical education settings where maintaining feedback quality is challenging.

Beyond these outcomes, the findings highlight important contributions. This is the first experimental study to systematically embed ChatGPT into RTS in physical education, offering a novel model that combines human interaction with AI-driven feedback. From a practical perspective, faculty can adopt ChatGPT-supported RTS in large-class PE settings to maintain feedback quality despite instructor constraints, thereby addressing one of the most persistent challenges in physical education instruction. Importantly, ChatGPT should be regarded as a complement to not a replacement for teachers, ensuring that AI-driven feedback enhances rather than substitutes for human expertise. This study therefore contributes to advancing sustainable, technology-enhanced practices in higher education, aligning with Saudi Vision 2030's priorities for digital innovation and pedagogical modernization. The findings suggest that ChatGPT can support feedback delivery in university physical education settings. Its accessibility may offer a practical option for instructors working with large groups. However, long-term sustainability and instructional impact require further investigation.

Responsible implementation of AI-supported feedback is essential. Effective use requires teacher training to ensure appropriate use, accurate interpretation of feedback, and alignment with instructional objectives. Clear institutional guidance is also necessary to regulate AI use, verify feedback accuracy, and manage potential risks such as over-reliance. When supported by human supervision, AI-based feedback can enhance learning while preserving instructional quality and academic responsibility.

## Limitations and future research

While the findings of this study provide strong evidence for the effectiveness of ChatGPT-supported Reciprocal Teaching Style (RTS), several limitations should be acknowledged.

First, students' attitudes were assessed using self-report questionnaires. Although the instrument demonstrated high internal consistency (*α* = .91), the very positive responses for enjoyment and motivation may have been influenced by social desirability or novelty effects. This may partially explain the elevated affective scores.

Second, all skill assessments were conducted within a single semester. The substantial improvements observed in defense, passing, and dribbling therefore do not confirm whether these gains would persist over time. Longitudinal research is needed to examine skill retention.

Third, although statistical power was high for all outcomes (≥.95), and effect sizes were substantial (e.g., composite *η*^2^ = .873), the sample was drawn from a single cohort at one institution. Strong internal validity does not automatically ensure broad external generalizability.

In addition, the study did not directly measure specific process variables that may explain how structured digital feedback influenced motor performance. Constructs such as perceived feedback quality during practice, attentional focus during execution, self efficacy in motor performance, and actual time on task were not independently assessed. Future research may examine these variables using validated measures to clarify the mechanisms linking instructional feedback to performance improvement.

Finally, the study focused exclusively on fundamental handball skills. While shooting, goalkeeping, and faking improved significantly, it remains unclear whether similar effects would emerge in advanced skills or other sports contexts.

These limitations suggest several directions for future research. Longitudinal studies should examine the durability of skill gains and attitudinal changes. Comparative studies may evaluate digital feedback alongside video analysis or motion tracking systems. Expanding research to different sports and educational settings would strengthen external validity. Mixed method designs may also provide deeper insight into students' learning experiences.

## Data Availability

The original contributions presented in the study are included in the article/Supplementary Material, further inquiries can be directed to the corresponding author/s.
