# Supplementary material for: Digital feedback via free ChatGPT within the reciprocal teaching style: improving fundamental handball skills and students' attitudes among university beginners
Source: Front Sports Act Living. 2026 Mar 25;8:1772502. doi: 10.3389/fspor.2026.1772502 (PMC13058805; doi:10.3389/fspor.2026.1772502)
Supplement: Supplementary file 1 [file Table1.docx]

Detailed Item-Total Correlations and Cronbach’s Alpha if Item Deleted for the Students’ Attitudes Scale (N = 48)

| Item | Statement | Item-Total Correlation (r) | Cronbach’s Alpha if Item Deleted |
| --- | --- | --- | --- |
| 1 | ChatGPT simplified the understanding of handball skills. | .50** | 0.86 |
| 2 | ChatGPT improved retention and comprehension. | .48** | 0.86 |
| 3 | ChatGPT provided feedback that helped correct mistakes. | .50** | 0.86 |
| 4 | ChatGPT made the lessons more meaningful. | .55** | 0.86 |
| 5 | ChatGPT feedback supported the achievement of objectives. | .63** | 0.86 |
| 6 | Using ChatGPT during lessons was enjoyable. | .36** | 0.87 |
| 7 | ChatGPT enriched my learning experience. | .47** | 0.86 |
| 8 | ChatGPT feedback increased my motivation to learn. | .58** | 0.86 |
| 9 | ChatGPT encouraged me to participate actively. | .36** | 0.87 |
| 10 | ChatGPT feedback increased my confidence. | .45** | 0.86 |
| 11 | ChatGPT supported peer-to-peer interaction. | .48** | 0.86 |
| 12 | ChatGPT improved peer observations. | .41** | 0.86 |
| 13 | I trust the accuracy of ChatGPT’s feedback. | .39** | 0.87 |
| 14 | ChatGPT feedback was consistent and reliable. | .49** | 0.86 |
| 15 | I would like to continue using ChatGPT. | .50** | 0.86 |
| 16 | I would recommend ChatGPT to others. | .41** | 0.86 |
| 17 | ChatGPT helped me evaluate my performance accurately. | .40** | 0.87 |
| 18 | ChatGPT feedback supported self-correction. | .24** | 0.87 |
| 19 | ChatGPT feedback improved my decision-making during practice. | .60** | 0.86 |
| 20 | ChatGPT feedback enhanced my overall learning process. | .48** | 0.86 |

Correlation is significant at the 0.05 level (2-tailed). *
** Correlation is significant at the 0.01 level (2-tailed).

Cronbach’s Alpha Coefficient for the Students’ Attitudes Scale (N = 48)

| Scale | Number of Items | Cronbach’s Alpha (α) |
| --- | --- | --- |
| Cognitive Domain | 5 | 0.87 |
| Affective Domain | 8 | 0.76 |
| Behavioral Domain | 7 | 0.77 |
| Overall Scale | 20 | 0.87 |
