# Supplementary material for: Digital feedback via free ChatGPT within the reciprocal teaching style: improving fundamental handball skills and students' attitudes among university beginners
Source: Front Sports Act Living. 2026 Mar 25;8:1772502. doi: 10.3389/fspor.2026.1772502 (PMC13058805; doi:10.3389/fspor.2026.1772502)
Supplement: Supplementary file 2 [file Table2.docx]

Item–Total Correlations and Descriptive Statistics for the Students’ Attitudes Scale (*N* = 48)

| Item | Statement | *M* | *SD* | Item-Total Correlation (r) |
| --- | --- | --- | --- | --- |
| 1 | ChatGPT simplified the understanding of handball skills. | 3.69 | 1.22 | .50** |
| 2 | ChatGPT improved retention and comprehension. | 3.65 | 1.28 | .48** |
| 3 | ChatGPT provided feedback that helped correct mistakes. | 3.77 | 1.17 | .50** |
| 4 | ChatGPT made the lessons more meaningful. | 3.77 | 1.21 | .55** |
| 5 | ChatGPT feedback supported the achievement of objectives. | 3.75 | 1.16 | .63** |
| 6 | Using ChatGPT during lessons was enjoyable. | 3.77 | 1.17 | .36** |
| 7 | ChatGPT enriched my learning experience. | 3.92 | 0.99 | .47** |
| 8 | ChatGPT feedback increased my motivation to learn. | 3.69 | 1.19 | .58** |
| 9 | ChatGPT encouraged me to participate actively. | 3.67 | 1.19 | .36** |
| 10 | ChatGPT feedback increased my confidence. | 3.83 | 1.1 | .45** |
| 11 | ChatGPT supported peer-to-peer interaction. | 3.69 | 1.15 | .48** |
| 12 | ChatGPT improved peer observations. | 3.81 | 1.08 | .41** |
| 13 | I trust the accuracy of ChatGPT’s feedback. | 3.81 | 1.07 | .39** |
| 14 | ChatGPT feedback was consistent and reliable. | 3.69 | 1.15 | .49** |
| 15 | I would like to continue using ChatGPT. | 3.71 | 1.17 | .50** |
| 16 | I would recommend ChatGPT to others. | 3.81 | 1.14 | .41** |
| 17 | ChatGPT helped me evaluate my performance accurately. | 3.73 | 1.12 | .40** |
| 18 | ChatGPT feedback supported self-correction. | 3.73 | 1.18 | .24** |
| 19 | ChatGPT feedback improved my decision-making during practice. | 3.79 | 1.11 | .60** |
| 20 | ChatGPT feedback enhanced my overall learning process. | 3.73 | 1.12 | .48** |

Correlation is significant at the 0.05 level (2-tailed). *
** Correlation is significant at the 0.01 level (2-tailed).
