# Supplementary material for: Digital feedback via free ChatGPT within the reciprocal teaching style: improving fundamental handball skills and students' attitudes among university beginners
Source: Front Sports Act Living. 2026 Mar 25;8:1772502. doi: 10.3389/fspor.2026.1772502 (PMC13058805; doi:10.3389/fspor.2026.1772502)
Supplement: Supplementary file 3 [file Datasheet1.pdf]

The 15-Week Educational Program

Teaching Units and Training Drills in Handball

Designed by
Prof. Dr. Ahmed Hassan Rakha
Professor of Teaching Methods in Physical Education

Table of Contents

Handball Skills and Drills Included in the Program ... *7*

A1 – Ball Familiarization ... **7**
- A1a – Basic Ball Handling ... 7
- A1b – Partner Familiarization ... 7
- A1c – Group Coordination ... 8

A2 – Ball Manipulation ... **8**
- A2a – Figure & Body Control ... 8

A3 – Catching ... **9**
- A3a – Chest Catch ... 9
- A3b – Overhead Catch ... 9
- A3c – Low Catch ... 9
- A3d – Jump Catch ... 9
- A3e – Running Catch ... 10

A4 – Passing ... **10**
- A4a – Chest Pass ... 10
- A4b – Bounce Pass ... 10
- A4c – Overhead Pass ... 11
- A4d – Quick Release Pass ... 11
- A4e – Bounce on the Move ... 11
- A4f – Lob Pass ... 11
- A4g – Give-and-Go ... 12
- A4h – Jump Pass ... 12
- A4i – Target Passing ... 12
- A4j – One-Handed Pass ... 12
- A4k – Fast-Break Pass ... 13
- A4l – No-Look Pass ... 13

A5 – Dribbling ... **13**
- A5a – Basic Dribble ... 13
- A5b – Change of Direction ... 14
- A5c – Speed Dribble ... 14
- A5d – Crossover Dribble ... 14
- A5e – Spin Dribble ... 14

A6 – Shooting ... **15**
- A6a – Overarm Shot ... 15
- A6b – Time-Limited Shot ... 15
- A6c – Falling Shot ... 15
- A6d – Underarm Shot ... 16

A6e – Jump & Rotate Shot 16
A6f – Fly Shot 16
A6g – Accuracy Shot 16
A6h – Lob Shot 17
A6i – Bounce Shot 17

A7 – Faking **17**
A7a – Passing Fake 17
A7b – Shooting Fake 17
A7c – Body Fake 18
A7d – Combined Fake 18

D – Defence **18**
D1 – Defensive Stance 18
D2 – On-Ball Defence 19
D3 – Interception 19
D4 – Pivot Defence 19
D5 – Block Shot 19
D6 – Help Defence 20
D7 – Switch Defence 20
D8 – Rotation Defence 20
D9 – Zone Coverage 20
D10 – Box-out 21
D11 – Legal Contact 21

G – Goalkeeping **21**
G1 – Ready Stance 21
G2 – Positioning 21
G3 – High Saves 22
G4 – Low Saves 22
G5 – Side Movement 22
G6 – Distribution 22

Integration & Small-Sided Games (SSG) **23**
SSG-D01: 3v3 Continuous 23
SSG-D02: 4v4 Transition 23
SSG-D03: 4v3 Overload 23
SSG-D04: 5v5 Half-Court 23
SSG-D05: Fun Mini-Games 23

The 15-Week Educational Program Schedule* *24

The Educational Program Teaching Units* *26

Teaching Unit 1 – Ball Familiarization* *26

1. Objectives 26
2. Lesson Phases 26
3. Reciprocal Style Implementation 27
4. Observation Checklist 27
Teaching Unit 2 – Moving Dribble & Advanced Ball Manipulation *28*
1. Objectives 28
2. Lesson Phases 28
3. Reciprocal Style Implementation 29
4. Observation Checklist 29
Teaching Unit 3 – Combined Dribbling & Chest Catch *30*
1. Objectives 30
2. Lesson Phases 30
3. Reciprocal Style Implementation 31
4. Observation Checklist 31
Teaching Unit 4 – Overhead Catch & Chest Pass *32*
1. Objectives 32
2. Lesson Phases 32
3. Reciprocal Style Implementation 33
4. Observation Checklist 33
Teaching Unit 5 – Low Catch & Bounce Pass *34*
1. Objectives 34
2. Lesson Phases 34
3. Reciprocal Style Implementation 35
4. Observation Checklist 35
Teaching Unit 6 – Jump Catch & Overhead Pass *36*
1. Objectives 36
2. Lesson Phases 36
3. Reciprocal Style Implementation 37
4. Observation Checklist 37
Teaching Unit 7 – Straight Dribble & Passing Revision *38*

1. Objectives 38
2. Lesson Phases 38
3. Reciprocal Style Implementation 39
4. Observation Checklist 39
Teaching Unit 8 – Zigzag Dribble & Passing Integration 40
1. Objectives 40
2. Lesson Phases 40
3. Reciprocal Style Implementation 41
4. Observation Checklist 41
Teaching Unit 9 – Protective Dribble & Catch/Pass Integration 42
1. Objectives 42
2. Lesson Phases 42
3. Reciprocal Style Implementation 43
4. Observation Checklist 43
Teaching Unit 10 – Speed Dribble & Overarm Shot 44
1. Objectives 44
2. Lesson Phases 44
3. Reciprocal Style Implementation 45
4. Observation Checklist 45
Teaching Unit 11 – Spin Dribble & Passing Fake 46
1. Objectives 46
2. Lesson Phases 46
3. Reciprocal Style Implementation 47
4. Observation Checklist 47
Teaching Unit 12 – Lob Shot, Bounce Shot & Shot Fake 48
1. Objectives 48
2. Lesson Phases 48
3. Reciprocal Style Implementation 49
4. Observation Checklist 49
Teaching Unit 13 – Rotation Shot, Body Fake & Intro to Defence 50

1. Objectives **50**
2. Lesson Phases.. 50
3. Reciprocal Style Implementation **51**
4. Observation Checklist **51**
Teaching Unit 14 – On-Ball Defence, Goalkeeping Basics & Small-Sided Game ***52***
1. Objectives **52**
2. Lesson Phases.. **52**
3. Reciprocal Style Implementation **53**
4. Observation Checklist **53**
Teaching Unit 15 – Zone Defence, Advanced Goalkeeping & Final Small-Sided Game 54
1. Objectives **54**
2. Lesson Phases.. **54**
3. Reciprocal Style Implementation **55**
4. Observation Checklist **55**

Handball Skills and Drills Included in the Program

A1 – Ball Familiarization

A1a – Basic Ball Handling

- **A1a-D01: Stationary Dribble**

  Setup: 1 ball per player, 3×3 m space each.

  Steps:

  1. Adopt athletic stance.
  2. Dribble with right hand 20–30 s.
  3. Switch to left hand.
  4. Alternate continuously.

     Key Points: Fingertips, eyes up, bounce at hip.

     Common Mistakes: Looking down, using palm, bounce too high.

- **A1a-D02: Dribble & Freeze**

  Setup: Open space, 1 ball per player.

  Steps:

  1. Dribble freely.
  2. On coach's whistle → stop & hold with 2 hands.
  3. Resume on signal.

     Key Points: Quick reaction, stable freeze.

     Common Mistakes: Slow stop, ball slips.

A1b – Partner Familiarization

- **A1b-D01: Partner Ball Games**

  Setup: Pairs 4–6 m apart, 1 ball.

  Steps: Exchange chest passes, bounce passes, overhead passes.

  Key Points: Communication, accuracy.

Mistakes: Weak passes, late hands.

A1c – Group Coordination

- **A1c-D01: Circle Exchange**

  Setup: 8–12 players in circle, 2 balls.

  Steps: Pass clockwise with ball A, counter-clockwise with ball B. Call name before pass.

  Key Points: Awareness, timing.

  Mistakes: Collisions, dropped balls.

A2 – Ball Manipulation

A2a – Figure & Body Control

- **A2a-D01: Figure-Eight Dribble**

  Setup: 1 ball per player.

  Steps: Wide stance, dribble between legs in ∞ pattern.

  Key Points: Smooth rhythm, eyes up.

  Mistakes: Jerky bounces, head down.

- **A2a-D02: Around-the-Body Dribble**

  Setup: 1 ball per player.

  Steps: Circle waist, knees, head with ball.

  Key Points: Controlled hands, steady tempo.

  Mistakes: Dropping ball, uneven rhythm.

- **A2a-D03: Crossover Dribble**

  Setup: Cones line, 1 ball per player.

  Steps: Dribble to cone, quick front cross, continue.

  Key Points: Protect ball, low stance.

Mistakes: Ball exposed, lost control.

A3 – Catching

A3a – Chest Catch

- **A3a-D01: Basic Chest Catch**

  Setup: Pairs 5 m apart.

  Steps: Pass chest pass → receiver cushions to chest.

  Key Points: Hands early, elbows soft.

  Mistakes: Rigid arms, rebound.

A3b – Overhead Catch

- **A3b-D01: Overhead Catch**

  Setup: Pairs 6–8 m.

  Steps: Receiver arms extended, catches ball high, brings down.

  Key Points: Meet ball early.

  Mistakes: Catch too close to face.

A3c – Low Catch

- **A3c-D01: Low Pocket Catch**

  Setup: Bounce pass drill.

  Steps: Bend knees, hands low pocket, absorb ball.

  Key Points: Use legs, not back.

  Mistakes: Back bending, stiff arms.

A3d – Jump Catch

- **A3d-D01: Jump & Secure**

  Setup: Lob from partner.

Steps: Approach, time jump, catch at peak, land balanced.

Key Points: Early jump, soft landing.

Mistakes: Late timing, off-balance.

A3e – Running Catch

- **A3e-D01: Catch on the Move**

  Setup: 12 m lane, moving pass.

  Steps: Sprint, show hands, catch in stride.

  Key Points: Don't slow down, absorb ball.

  Mistakes: Stopping before catch.

A4 – Passing

A4a – Chest Pass

- **A4a-D01: Direct Chest Pass**

  Setup: Pairs 6 m apart.

  Steps: Hold ball at chest → step → extend arms → snap wrists.

  Key Points: Direct, accurate, follow-through.

  Mistakes: No step, weak power.

A4b – Bounce Pass

- **A4b-D01: Bounce to Teammate**

  Setup: Mark bounce point ⅔ distance.

  Steps: Push ball firmly to floor.

  Key Points: Correct force, timing.

  Mistakes: Bounce too near/far.

A4c – Overhead Pass

- **A4c-D01: Long Overhead Pass**

  Setup: 8–10 m pairs.

  Steps: Raise ball overhead, step, throw high arc.

  Key Points: Strong arms, trajectory.

  Mistakes: Flat pass.

A4d – Quick Release Pass

- **A4d-D01: Catch-and-Fire**

  Setup: Triangle 3 players.

  Steps: Catch ball → immediate pass to 3rd.

  Key Points: Speed, anticipation.

  Mistakes: Hesitation, bad accuracy.

A4e – Bounce on the Move

- **A4e-D01: Jogging Bounce Pass**

  Setup: Pairs running parallel.

  Steps: Bounce pass in stride.

  Key Points: Lead teammate.

  Mistakes: Pass behind feet.

A4f – Lob Pass

- **A4f-D01: Over-the-Top Pass**

  Setup: Cone/defender between players.

  Steps: High arc over obstacle.

  Key Points: Soft release, timing.

Mistakes: Too low lob.

A4g – Give-and-Go

- **A4g-D01: Pass, Cut, Return**

  Setup: 2 players, cone cut zone.

  Steps: Pass → cut fast → receive return pass.

  Key Points: Speed after pass.

  Mistakes: Slow cut, late return.

A4h – Jump Pass

- **A4h-D01: Pass in the Air**

  Setup: Pairs 6 m apart.

  Steps: Jump → release ball mid-air → land controlled.

  Key Points: Timing, balance.

  Mistakes: Late release.

A4i – Target Passing

- **A4i-D01: Pass to Spots**

  Setup: 4 wall targets.

  Steps: Coach calls target, player passes instantly.

  Key Points: Accuracy, reaction.

  Mistakes: Missed target.

A4j – One-Handed Pass

- **A4j-D01: Wrist Snap Pass**

  Setup: Pairs 5 m.

  Steps: Quick wrist release.

Key Points: Speed, accuracy.

Mistakes: Side spin.

A4k – Fast-Break Pass

- **A4k-D01: Outlet to Runner**

  Setup: GK/feeder + sprinting wing.

  Steps: Secure ball → long pass into space.

  Key Points: Lead runner.

  Mistakes: Over/under-shoot.

A4l – No-Look Pass

- **A4l-D01: Deceptive Feed**

  Setup: Triangle with defender.

  Steps: Look away, pass to actual receiver.

  Key Points: Sell fake with eyes.

  Mistakes: Telegraphing.

A5 – Dribbling

A5a – Basic Dribble

- **A5a-D01: Straight Dribble**

  Setup: 15 m lane.

  Steps: Dribble forward dominant hand, return weak hand.

  Key Points: Eyes up, controlled bounce.

  Mistakes: Looking down, bounce too high.

A5b – Change of Direction

- **A5b-D01: Zig-Zag Dribble**

  Setup: Cones 2 m apart.

  Steps: Dribble to cone → crossover → continue.

  Key Points: Low stance, plant foot.

  Mistakes: Ball exposed.

A5c – Speed Dribble

- **A5c-D01: Full Court Sprint Dribble**

  Setup: Baseline to baseline.

  Steps: Push ball ahead, sprint in control.

  Key Points: Push-catch rhythm.

  Mistakes: Ball too far, loss of control.

A5d – Crossover Dribble

- **A5d-D01: Front Cross Rhythm**

  Setup: Open space.

  Steps: Two bounces R, quick cross to L, repeat.

  Key Points: Quick snap, protect ball.

  Mistakes: Slow execution.

A5e – Spin Dribble

- **A5e-D01: 360 Spin**

  Setup: Cone as defender.

  Steps: Approach → plant → spin back to defender → exit dribble.

  Key Points: Stay low, tight control.

Mistakes: Off-balance, losing ball

A6 – Shooting

A6a – Overarm Shot

- **A6a-D01: Stationary Overarm Shot**

  Setup: Shooter at 6–7 m, feeder, GK optional.

  Steps:

  1. Catch in balanced stance.
  2. Step with non-throwing foot.
  3. Raise elbow high, ball behind head.
  4. Snap wrist, follow through to target.

     Key Points: Eyes on corner, transfer weight.

     Mistakes: Dropping elbow, weak wrist.

A6b – Time-Limited Shot

- **A6b-D01: 3-Second Release**

  Setup: Timer + feeder.

  Steps: Catch ball → shoot within 3 seconds.

  Key Points: Fast decision-making.

  Mistakes: Rushing with no aim, late release.

A6c – Falling Shot

- **A6c-D01: Forward Lean Shot**

  Setup: Shooter starts close to 6 m.

  Steps: Drive forward → lean body → release before feet land.

  Key Points: Balance, quick release.

  Mistakes: Shooting too late after landing.

A6d – Underarm Shot

- **A6d-D01: Low Whip Shot**

  Setup: 1 ball/player.

  Steps: Arm swings low, release ball close to ground.

  Key Points: Shoulder drives forward, eyes on low target.

  Mistakes: Weak release, off-target.

A6e – Jump & Rotate Shot

- **A6e-D01: Jump Turn Shot**

  Setup: Shooter jumps near 6 m.

  Steps: Jump → rotate 90–180° mid-air → shoot before landing.

  Key Points: Control in air, aim before release.

  Mistakes: Poor balance, late shot.

A6f – Fly Shot

- **A6f-D01: Catch-Jump-Shoot**

  Setup: Passer feeds to attacker jumping into goal area.

  Steps: Catch in air → release before landing inside 6 m.

  Key Points: Timing with pass, shoot mid-flight.

  Mistakes: Landing before release → foul.

A6g – Accuracy Shot

- **A6g-D01: Target Corners**

  Setup: Cones in goal corners.

  Steps: Shoot to hit specific target zones.

Key Points: Focus, precision.

Mistakes: Power over accuracy.

A6h – Lob Shot

- **A6h-D01: High Arc Finish**

  Setup: GK present.

  Steps: Approach → lift elbow → high arc over GK.

  Key Points: Timing release, soft touch.

  Mistakes: Too low arc, GK catches.

A6i – Bounce Shot

- **A6i-D01: One-Bounce Finish**

  Setup: Shooter at 7 m.

  Steps: Throw to bounce 2–3 m before goal line.

  Key Points: Ball should rise unpredictably.

  Mistakes: Bounce too early or late.

A7 – Faking

A7a – Passing Fake

- **A7a-D01: Fake Pass, Drive**

  Setup: 1v0 → add defender later.

  Steps: Show strong pass motion → suddenly drive opposite.

  Key Points: Convincing body/eye fake, fast follow-up.

  Mistakes: Weak fake, hesitation.

A7b – Shooting Fake

- **A7b-D01: Pump Fake Shot**

Setup: Player at 9 m.

Steps: Raise arm as if shooting → pull ball back → pass or dribble.

Key Points: Shoulder lift, sell shot.

Mistakes: Too slow, defender doesn't bite.

A7c – Body Fake

- **A7c-D01: Side Step Fake**

  Setup: Cone as defender.

  Steps: Lean shoulders one way → quick push opposite.

  Key Points: Strong lean, quick recovery.

  Mistakes: Weak lean, slow execution.

A7d – Combined Fake

- **A7d-D01: Double Fake Combo**

  Setup: Attacker with ball, passive defender.

  Steps: Pass fake → shot fake → real action.

  Key Points: Fast sequence, maintain control.

  Mistakes: Overdoing → loss of timing.

D – Defence

D1 – Defensive Stance

- **D1-D01: Basic Stance & Slide**

  Setup: 1v0 lanes.

  Steps: Knees bent, arms wide → slide left/right → recover stance.

  Key Points: Low hips, eyes on attacker.

  Mistakes: Crossing feet, standing tall.

D2 – On-Ball Defence

- **D2-D01: Mirror Drill**

  Setup: Attacker dribbles, defender mirrors.

  Steps: Stay square, arms active, cut off drives.

  Key Points: Chest to ball, no reaching.

  Mistakes: Fouling, slow feet.

D3 – Interception

- **D3-D01: Passing Lane Read**

  Setup: Feeder passes, defender anticipates.

  Steps: Watch passer → jump lane → intercept with both hands.

  Key Points: Timing, anticipation.

  Mistakes: Early step, missed ball.

D4 – Pivot Defence

- **D4-D01: Deny Pivot**

  Setup: Attacker acts pivot.

  Steps: Maintain body contact, front pivot when ball comes.

  Key Points: Contact early, active arms.

  Mistakes: Losing position.

D5 – Block Shot

- **D5-D01: Jump Block**

  Setup: Shooter vs defender.

  Steps: Defender times jump to block.

  Key Points: Arms vertical, jump straight up.

Mistakes: Leaning forward, fouling.

D6 – Help Defence

- **D6-D01: Help & Recover**

  Setup: 2 attackers vs 2 defenders.

  Steps: Defender helps vs dribble → sprints back to own player.

  Key Points: Quick recovery, communication.

  Mistakes: Late closeout.

D7 – Switch Defence

- **D7-D01: Screen Switch**

  Setup: 2v2 with screen.

  Steps: Call switch early, exchange marks.

  Key Points: Loud call, timing.

  Mistakes: Both follow same man.

D8 – Rotation Defence

- **D8-D01: 3-Man Rotation**

  Setup: Attack overload.

  Steps: First defender helps, others rotate to cover.

  Key Points: Communication, awareness.

  Mistakes: Late rotations.

D9 – Zone Coverage

- **D9-D01: 6:0 Zone Drill**

  Setup: 6 defenders vs attack.

  Steps: Defenders shift as a unit with ball.

Key Points: Compact line, step forward on shooter.

Mistakes: Gaps between defenders.

D10 – Box-out

- **D10-D01: Rebound Control**

  Setup: Shots at goal, defenders box-out.

  Steps: Hit, find, hold attacker, secure rebound.

  Key Points: Early contact, wide base.

  Mistakes: Watching ball only, losing man.

D11 – Legal Contact

- **D11-D01: Contain without Foul**

  Setup: 1v1 attacker vs defender.

  Steps: Use chest, arms wide, avoid reaching.

  Key Points: Strong stance, body not hands.

  Mistakes: Excess hand use → fouls.

G – Goalkeeping

G1 – Ready Stance

- **G1-D01: Set Position Drill**

  Setup: GK center of goal, feeder at 9 m.

  Steps: Small steps, knees bent, hands forward.

  Key Points: Stay light, balanced.

G2 – Positioning

- **G2-D01: Angle Movement**

  Setup: GK tracks ball side to side.

Steps: Move on arc, adjust angles.

Key Points: Body centered.

G3 – High Saves

- **G3-D01: Top Corner Stops**

  Setup: Shooter aims high corners.

  Steps: Extend arms, parry or catch.

  Key Points: Don't lean back.

G4 – Low Saves

- **G4-D01: Low Dive**

  Setup: Shooter aims low.

  Steps: Drop/knee bend, hands quick to floor.

  Key Points: Body behind ball.

G5 – Side Movement

- **G5-D01: Shuffle Drill**

  Setup: Feeder passes ball side-to-side.

  Steps: Quick shuffle, set before shot.

  Key Points: Small steps, balanced stance.

G6 – Distribution

- **G6-D01: Outlet Throw**

  Setup: GK saves, then throws to wing runner.

  Steps: Quick scan, long pass into stride.

  Key Points: Lead pass, start fast break.

Integration & Small-Sided Games (SSG)

SSG-D01: 3v3 Continuous

Objective: Quick decision-making, transition.

Rules: Score after ≥3 passes, losers sprint to endline.

Key Points: Width, depth, communication.

SSG-D02: 4v4 Transition

Objective: Defence-to-attack speed.

Rules: On turnover, 5 s to cross midline.

SSG-D03: 4v3 Overload

Objective: Attack exploit, defence adapt.

Key Points: Quick pass, rotations.

SSG-D04: 5v5 Half-Court

Objective: Apply tactics, set plays.

Rules: 30 s possession limit.

SSG-D05: Fun Mini-Games

Objective: Intensity + motivation.

Format: First to 3 goals wins, touch limits optional.

The 15-Week Educational Program Schedule

Weeks	Skills & Codes	Drills & Codes (Examples)
**Weeks 1–3**	**A1: Ball Familiarization** (A1a, A1b, A1c)<br>**A2: Ball Manipulation** (A2a, A2b)<br>Intro **A3/A4: Catching & Passing**	A1a-D01: Stationary Dribble<br>A1a-D02: Dribble & Freeze<br>A2a-D01: Figure-Eight Dribble<br>A2a-D02: Around Waist & Legs<br>A1b-D01: Walking Dribble<br>A1b-D02: Jogging Dribble<br>A1c-D01: Stationary + Moving Combo<br>A3a-D01: Basic Chest Catch<br>A4a-D01: Direct Chest Pass
**Weeks 4–6**	**A3: Catching** (A3b, A3c, A3d)<br>**A4: Passing** (A4a–A4c) + review A1–A2	A3b-D01: Overhead Catch Static<br>A3c-D01: Low Catch Solo<br>A3d-D01: Jump Catch Solo<br>A4a-D02: Quick Chest Pass<br>A4b-D01: Bounce Pass Static<br>A4b-D02: Bounce Pass on Move<br>A4c-D01: Overhead Pass Static<br>A4c-D02: Overhead Pass on Move
**Weeks 7–9**	**A5: Dribbling** (A5a–A5c) + continuation A3–A4	A5a-D01: Dribble Straight Line<br>A5a-D02: Dribble with Partner Pressure<br>A5b-D01: Zigzag Cones Dribble<br>A5b-D02: Dribble with Defender Pressure<br>A5c-D01: Shielded Dribble Static<br>A5c-D02: Dribble with Defender Contact<br>A3a-D02: Catch in Pairs<br>A4b-D01: Bounce Pass Static

Weeks	Skills & Codes	Drills & Codes (Examples)
**Weeks 10–11**	**A6: Shooting** (A6a–A6c)<br>**A7: Faking** (A7a, A7b) + integration with A3–A5	A6a-D01: Overarm Shot Static<br>A6a-D02: Overarm Shot with Step<br>A6b-D01: Lob Shot Static<br>A6c-D01: Bounce Shot Solo<br>A7a-D01: Chest Pass Fake Static<br>A7b-D01: Overarm Shot Fake<br>A7b-D02: Fake then Dribble/Shot
**Weeks 12–13**	**A6d: Rotation Shot**<br>**A7c: Body Fake**<br>**D1–D2: Defence Basics**<br>**D3: On-Ball Defence**<br>**G1: Goalkeeping Basics**	A6d-D01: Rotation Shot Solo<br>A7c-D01: Body Fake Static<br>D1-D01: On-Ball Stance Drill<br>D2-D01: Help Defence Drill<br>D3-D01: 1v1 Defence Drill<br>D3-D02: Mirror Shuffle Drill<br>G1-D01: Basic Save Drill<br>G1-D02: Reaction Ball Drill
**Weeks 14–15**	**D7: Zone & Switch Defence**<br>**G4: Advanced Goalkeeping**<br>**SSG: Small-Sided Games**	D7-D01: Zone Drill<br>D7-D02: Switch Drill<br>G4-D01: Angle Save Drill<br>G4-D02: Diving Save Drill<br>SSG-D01: 3v3 Half Court<br>SSG-D02: 3v3 Transition Game<br>SSG-D03: 5v5 Half Court<br>SSG-D04: 5v5 Transition Game

The Educational Program Teaching Units

Teaching Unit 1 – Ball Familiarization

Date: 22 Jan 2025
Duration: 90 minutes
Focus Skills: A1a – Basic Ball Handling, A2a – Ball Manipulation

1. Objectives

- **Cognitive:** Identify rules of safe ball handling and ball manipulation.
- **Skill:** Perform stationary dribble and figure-eight ball control.
- **Physical:** Improve coordination, rhythm, and balance.
- **Affective:** Build confidence and enjoyment when using the ball.

2. Lesson Phases

A. Warm-Up (10 minutes)

- Light jogging with ball, dynamic stretches.
- Partner tossing and catching games for activation.

B. Physical Preparation (10 minutes)

- Ladder drills for coordination.
- Quick-feet drills holding the ball.

C. Main Skill Development (50 minutes) – Reciprocal Style

- Students work in pairs (Performer + Observer).
- Teacher provides task cards with criteria for each drill.
- After each drill (5–7 minutes), roles are switched.

Skills & Drills:

- **A1a-D01: Stationary Dribble** – Dribble at waist height using fingertips, eyes up.
- **A1a-D02: Dribble & Freeze** – Dribble continuously, freeze on teacher's signal.
- **A2a-D01: Figure-Eight Dribble** – Move ball between legs in a figure-eight motion.
- **A2a-D02: Around Waist & Legs** – Circle the ball around the waist and knees.

D. Application Game (15 minutes)

- Relay races: Teams dribble or perform figure-eight before tagging next player.

E. Cool-Down (5 minutes)

- Static stretching with ball.
- Relaxation dribbling with eyes closed (slow rhythm).

3. Reciprocal Style Implementation

- **Performer:** Executes the drill focusing on setup, steps, and key points.
- **Observer:** Uses observation checklist to evaluate performer against criteria.
- **Feedback:** Observer gives immediate positive/constructive feedback.
- **Switch Roles:** After drill, performer becomes observer.
- **Teacher's Role:** Supervises, clarifies, and provides corrective feedback when needed.

4. Observation Checklist

Skill Code	Drill	Key Performance Criteria	Observer Notes
A1a-D01	Stationary Dribble	• Uses fingertips not palm<br>• Dribble at waist height<br>• Eyes up<br>• Maintains control	
A1a-D02	Dribble & Freeze	• Responds to signal quickly<br>• Maintains ball control<br>• Balance preserved	
A2a-D01	Figure-Eight Dribble	• Smooth continuous motion<br>• Ball close to body<br>• Eyes forward	
A2a-D02	Around Waist & Legs	• Circles are smooth<br>• No ball drops<br>• Rhythm maintained	

Teaching Unit 2 – Moving Dribble & Advanced Ball Manipulation

Date: 29 Jan 2025
Duration: 90 minutes
Focus Skills: A1b – Moving Dribble, A2b – Advanced Manipulation

1. Objectives

- **Cognitive:** Recognize the principles of dribbling while moving and advanced manipulation patterns.
- **Skill:** Perform walking/jogging dribbles and crossover/body-wrap control.
- **Physical:** Improve agility, coordination, and dynamic balance.
- **Affective:** Develop persistence, enjoyment, and willingness to experiment with skills.

2. Lesson Phases

A. Warm-Up (10 minutes)

- Jogging around the court while bouncing the ball lightly.
- Dynamic stretching with ball (arm swings holding ball, torso twists with ball).

B. Physical Preparation (10 minutes)

- Shuttle runs while dribbling at slow pace.
- Quick directional changes (side steps, backpedal) without losing ball control.

C. Main Skill Development (50 minutes) – Reciprocal Style

- Students in pairs: one Performer, one Observer.
- Teacher distributes observation cards with criteria.
- Roles switch every drill (5–7 minutes).

Skills & Drills:

- **A1b-D01: Walking Dribble** – Dribble while walking, eyes up, controlled bounce.
- **A1b-D02: Jogging Dribble** – Increase speed, maintain rhythm, no looking down.
- **A1b-D03: Zigzag Dribble** – Dribble through cones with sharp direction changes.
- **A2b-D01: Crossover Dribble** – Switch ball from one hand to another in front of body.
- **A2b-D02: Body Wrap** – Circle ball around torso smoothly.
- **A2b-D03: Low Dribble Switch** – Alternate low bounces between hands.

D. Application Game (15 minutes)

- “Dribble & Tag”: Players dribble continuously; tagged player must perform crossover before continuing.

E. Cool-Down (5 minutes)

- Static stretching: focus on calves, thighs, shoulders.
- Slow stationary dribbling with breathing exercises.

3. Reciprocal Style Implementation

- **Performer:** Executes moving dribble or manipulation drill.
- **Observer:** Monitors performance using observation checklist.
- **Feedback:** Immediate peer feedback (praise + one correction).
- **Switch Roles:** Every 5–7 minutes.
- **Teacher’s Role:** Guide feedback, correct errors, ensure safety.

4. Observation Checklist

Skill Code	Drill	Key Performance Criteria	Observer Notes
A1b-D01	Walking Dribble	• Dribbles at hip height<br>• Eyes forward<br>• Maintains control	
A1b-D02	Jogging Dribble	• Maintains rhythm<br>• Doesn’t look down<br>• Controls ball near body	
A1b-D03	Zigzag Dribble	• Sharp change of direction<br>• Ball close to body<br>• Quick recovery after cone	
A2b-D01	Crossover Dribble	• Switches hands smoothly<br>• Maintains balance<br>• Eyes up	
A2b-D02	Body Wrap	• Smooth circles<br>• No ball drops<br>• Rhythm maintained	
A2b-D03	Low Dribble Switch	• Ball stays low<br>• Alternates hands quickly<br>• Eyes up	

Teaching Unit 3 – Combined Dribbling & Chest Catch

Date: 5 Feb 2025
Duration: 90 minutes
Focus Skills: A1c – Combined Dribbling, A3a – Chest Catch

1. Objectives

- **Cognitive:** Consolidate knowledge of dribbling variations and catching fundamentals.
- **Skill:** Perform combined dribbling patterns and execute chest catch with accuracy.
- **Physical:** Develop endurance, coordination, and reaction speed.
- **Affective:** Build trust between teammates and increase self-confidence.

2. Lesson Phases

A. Warm-Up (10 minutes)

- General jogging with ball.
- Dynamic stretches while bouncing or tossing the ball (arm swings, trunk twists).

B. Physical Preparation (10 minutes)

- Agility ladder runs with ball dribbling.
- Sprint and stop drills with quick ball control.

C. Main Skill Development (50 minutes) – Reciprocal Style

- Students work in pairs (Performer + Observer).
- Teacher distributes task cards with detailed criteria.
- Performers execute; observers assess using checklist.
- Switch roles after each drill.

Skills & Drills:

- **A1c-D01: Stationary + Moving Combo** – Alternate between static dribble and moving dribble.
- **A1c-D02: Left/Right Switch** – Switch smoothly between left and right hand while moving.
- **A3a-D01: Basic Chest Catch** – Receive ball with hands extended, absorb into chest.
- **A3a-D02: Catch in Pairs** – Partner passing and receiving at chest height.

D. Application Game (15 minutes)

- “Pass & Dribble Relay”: Player dribbles half court, passes to partner, who catches and dribbles back.

E. Cool-Down (5 minutes)

- Light jogging with ball.
- Static stretches focusing on shoulders, arms, and legs.

3. Reciprocal Style Implementation

- **Performer:** Executes combined dribble or chest catch.
- **Observer:** Uses observation checklist to track performer’s accuracy and control.
- **Feedback:** Observer provides immediate, constructive comments.
- **Switch Roles:** After each drill (5–7 minutes).
- **Teacher’s Role:** Clarify criteria, support peer assessment, correct major mistakes.

4. Observation Checklist

Skill Code	Drill	Key Performance Criteria	Observer Notes
A1c-D01	Stationary + Moving Combo	• Alternates smoothly<br>• Maintains rhythm<br>• Eyes up	
A1c-D02	Left/Right Switch	• Switches hand correctly<br>• Maintains control<br>• Dribbles with rhythm	
A3a-D01	Basic Chest Catch	• Hands shown early<br>• Elbows bend to absorb<br>• Ball secured to chest	
A3a-D02	Catch in Pairs	• Accurate partner passing<br>• Secure reception<br>• Maintains eye contact with ball	

Teaching Unit 4 – Overhead Catch & Chest Pass

Date: 12 Feb 2025
Duration: 90 minutes
Focus Skills: A3b – Overhead Catch, A4a – Chest Pass

1. Objectives

- **Cognitive:** Recognize the technique and cues of overhead catching and chest passing.
- **Skill:** Perform accurate overhead catches and chest passes with partner.
- **Physical:** Develop upper-body coordination, strength, and timing.
- **Affective:** Build self-confidence and cooperation between teammates.

2. Lesson Phases

A. Warm-Up (10 minutes)

- General jogging with ball around court.
- Partner tossing drills (light overhead throws and catches).
- Dynamic stretches focusing on arms and shoulders.

B. Physical Preparation (10 minutes)

- Medicine ball throws (lightweight) to strengthen arms.
- Quick-reaction partner taps (react to partner's overhead clap while holding ball).

C. Main Skill Development (50 minutes) – Reciprocal Style

- Students paired: one Performer, one Observer.
- Teacher provides observation cards with performance criteria.
- Performer executes drills, Observer records performance.
- Roles switch every 5–7 minutes.

Skills & Drills:

- **A3b-D01: Overhead Catch Static** – Catch ball thrown directly overhead while standing.
- **A3b-D02: Overhead Catch in Pairs** – Partner throws overhead, receiver absorbs ball safely.
- **A4a-D01: Direct Chest Pass** – Pass from chest directly to partner's chest.
- **A4a-D02: Quick Chest Pass** – Rapid successive chest passes with partner.

D. Application Game (15 minutes)

- “Pass & Catch Circle”: Students form circle, pass overhead or chest pass around with quick rotation.

E. Cool-Down (5 minutes)

- Stretching focusing on shoulders, arms, chest.
- Relaxed ball tossing between partners.

3. Reciprocal Style Implementation

- **Performer:** Executes overhead catch or chest pass.
- **Observer:** Uses observation checklist to assess criteria (stance, timing, accuracy).
- **Feedback:** Immediate, constructive peer feedback.
- **Switch Roles:** After each drill.
- **Teacher’s Role:** Supervises feedback process, ensures accuracy, reinforces correct technique.

4. Observation Checklist

Skill Code	Drill	Key Performance Criteria	Observer Notes
A3b-D01	Overhead Catch Static	• Hands above head early<br>• Elbows flex to absorb<br>• Eyes track ball	
A3b-D02	Overhead Catch in Pairs	• Catches securely<br>• Controls landing stance<br>• Communicates with partner	
A4a-D01	Direct Chest Pass	• Step with opposite foot<br>• Pass direct and accurate<br>• Follow-through with wrists	
A4a-D02	Quick Chest Pass	• Maintains rhythm<br>• Passes fast but accurate<br>• Keeps elbows high	

Teaching Unit 5 – Low Catch & Bounce Pass

Date: 19 Feb 2025
Duration: 90 minutes
Focus Skills: A3c – Low Catch, A4b – Bounce Pass

1. Objectives

- **Cognitive:** Identify correct technique and situations for low catching and bounce passing.
- **Skill:** Perform low catch with control and execute accurate bounce passes.
- **Physical:** Improve flexibility, reaction speed, and arm coordination.
- **Affective:** Build persistence, cooperation, and trust with teammates.

2. Lesson Phases

A. Warm-Up (10 minutes)

- Jogging with ball held low.
- Dynamic stretches: hamstring bends, lunges with ball.
- Partner ball rolling games (roll and catch low).

B. Physical Preparation (10 minutes)

- Quick side-to-side movement while reaching down to touch cones.
- Reaction drills: coach rolls ball unexpectedly, player catches it low.

C. Main Skill Development (50 minutes) – Reciprocal Style

- Students pair up: one **Performer**, one **Observer**.
- Teacher provides observation checklist with performance criteria.
- Performers execute drills; observers record and give feedback.
- Switch roles after each drill (every 5–7 minutes).

Skills & Drills:

- **A3c-D01: Low Catch Solo** – Roll ball against wall, player catches it low.
- **A3c-D02: Low Catch in Pairs** – Partner rolls ball, receiver bends and secures it.
- **A4b-D01: Bounce Pass Static** – Partner passes ball with bounce, receiver catches at chest.
- **A4b-D02: Bounce Pass on Move** – Player dribbles, executes bounce pass to moving partner.

D. Application Game (15 minutes)

- "Bounce & Catch Relay": Teams pass ball using only bounce passes; partner must perform low catch before continuing.

E. Cool-Down (5 minutes)

- Stretching focusing on hamstrings, back, shoulders.
- Slow relaxed passing between partners.

3. Reciprocal Style Implementation

- **Performer:** Executes low catch or bounce pass.
- **Observer:** Tracks accuracy, body position, and reaction using checklist.
- **Feedback:** Immediate peer feedback (positive + correction).
- **Switch Roles:** After each drill.
- **Teacher's Role:** Ensure criteria are applied consistently, provide corrective guidance.

4. Observation Checklist

Skill Code	Drill	Key Performance Criteria	Observer Notes
A3c-D01	Low Catch Solo	• Hands low and ready<br>• Bends knees not back<br>• Ball secured cleanly	
A3c-D02	Low Catch in Pairs	• Reacts quickly<br>• Absorbs ball into chest<br>• Maintains balance	
A4b-D01	Bounce Pass Static	• Ball bounces ⅔ distance<br>• Accurate to partner<br>• Step forward with pass	
A4b-D02	Bounce Pass on Move	• Pass timed correctly<br>• Ball easy to catch<br>• Maintains speed and accuracy	

Teaching Unit 6 – Jump Catch & Overhead Pass

Date: 5 Mar 2025
Duration: 90 minutes
Focus Skills: A3d – Jump Catch, A4c – Overhead Pass

1. Objectives

- **Cognitive:** Understand the technique and timing for jump catching and overhead passing.
- **Skill:** Perform accurate jump catches and execute overhead passes to teammates.
- **Physical:** Improve vertical jump, arm power, and upper-body coordination.
- **Affective:** Build confidence in contested situations and promote cooperation.

2. Lesson Phases

A. Warm-Up (10 minutes)

- Jogging with ball, include skipping and high knees.
- Dynamic stretches focusing on shoulders, arms, and legs.
- Light tossing of ball overhead in pairs to activate reaction.

B. Physical Preparation (10 minutes)

- Vertical jump practice without ball.
- Medicine ball overhead throws for power.

C. Main Skill Development (50 minutes) – Reciprocal Style

- Students in pairs (Performer + Observer).
- Teacher provides observation cards with performance criteria.
- Performers execute drills; observers record feedback.
- Switch roles every 5–7 minutes.

Skills & Drills:

- **A3d-D01: Jump Catch Solo** – Player tosses ball up, jumps, catches above head, and lands balanced.
- **A3d-D02: Jump Catch in Pairs** – Partner throws high ball, receiver times jump to catch it.
- **A4c-D01: Overhead Pass Static** – Player passes ball overhead to partner while stationary.
- **A4c-D02: Overhead Pass on Move** – Player runs forward and executes overhead pass.

D. Application Game (15 minutes)

- “High Ball Challenge”: Teams throw overhead passes; partner must jump catch before passing to next teammate.

E. Cool-Down (5 minutes)

- Gentle jogging, static stretches focusing on shoulders, arms, and calves.
- Partner relaxed toss and catch.

3. Reciprocal Style Implementation

- **Performer:** Executes jump catch or overhead pass.
- **Observer:** Monitors criteria such as timing, body control, and accuracy.
- **Feedback:** Observer provides immediate positive and corrective comments.
- **Switch Roles:** After each drill.
- **Teacher’s Role:** Guide feedback, ensure correct landing mechanics, and reinforce safe technique.

4. Observation Checklist

Skill Code	Drill	Key Performance Criteria	Observer Notes
A3d-D01	Jump Catch Solo	• Times jump correctly<br>• Hands extended above head<br>• Ball secured into chest<br>• Controlled landing	
A3d-D02	Jump Catch in Pairs	• Jumps at right moment<br>• Catches cleanly<br>• Maintains balance	
A4c-D01	Overhead Pass Static	• Holds ball above head<br>• Steps forward<br>• Pass is strong and accurate	
A4c-D02	Overhead Pass on Move	• Executes pass while running<br>• Maintains control<br>• Pass reaches partner accurately	

Teaching Unit 7 – Straight Dribble & Passing Revision

Date: 12 Mar 2025
Duration: 90 minutes
Focus Skills: A5a – Straight Dribble, Revision of A4 (Passing)

1. Objectives

- **Cognitive:** Recognize when to use straight dribble in attack and recall chest/bounce pass principles.
- **Skill:** Perform controlled forward dribble and accurate chest/bounce passes.
- **Physical:** Improve running coordination, arm–leg rhythm, and endurance.
- **Affective:** Build confidence in advancing the ball and strengthen cooperation with teammates.

2. Lesson Phases

A. Warm-Up (10 minutes)

- Jogging with ball in hand.
- Dynamic stretches: high knees, butt kicks, arm swings while holding ball.
- Simple passing in pairs at jogging pace.

B. Physical Preparation (10 minutes)

- Straight-line sprints while dribbling at slow pace.
- Cone running drills with ball control.

C. Main Skill Development (50 minutes) – Reciprocal Style

- Students paired: one **Performer**, one **Observer**.
- Teacher provides task cards with performance criteria.
- Roles switch every 5–7 minutes.

Skills & Drills:

- **A5a-D01: Dribble Straight Line** – Dribble in straight path, eyes up, control ball.
- **A5a-D02: Dribble with Partner Pressure** – Advance dribble with light pressure from partner.
- **A5a-D03: Dribble to Cone** – Sprint while dribbling, stop control at cone.
- **A4a-D01: Direct Chest Pass** – Accurate chest pass to partner after dribble.
- **A4b-D01: Bounce Pass Static** – Bounce pass to partner with correct timing.

D. Application Game (15 minutes)

- "Dribble & Pass Relay": Players dribble to half court, pass to partner, who continues the sequence.

E. Cool-Down (5 minutes)

- Gentle jogging, stretching legs and arms.
- Partner relaxed passes to end session.

3. Reciprocal Style Implementation

- **Performer:** Executes straight dribble or pass.
- **Observer:** Uses checklist to monitor stance, control, and accuracy.
- **Feedback:** Observer provides quick positive feedback + one corrective note.
- **Switch Roles:** After each drill.
- **Teacher's Role:** Supervise peer feedback, reinforce correct dribbling and passing mechanics.

4. Observation Checklist

Skill Code	Drill	Key Performance Criteria	Observer Notes
A5a-D01	Dribble Straight Line	• Ball close to body<br>• Eyes up<br>• Maintains rhythm	
A5a-D02	Dribble with Partner Pressure	• Protects ball with body<br>• Maintains control under pressure<br>• Eyes forward	
A5a-D03	Dribble to Cone	• Controls ball while sprinting<br>• Stops at cone<br>• Ball not lost	
A4a-D01	Direct Chest Pass	• Step with opposite foot<br>• Pass straight to partner<br>• Follow-through	
A4b-D01	Bounce Pass Static	• Ball bounces at ⅔ distance<br>• Accurate to partner<br>• Quick release	

Teaching Unit 8 – Zigzag Dribble & Passing Integration

Date: 19 Mar 2025
Duration: 90 minutes
Focus Skills: A5b – Zigzag Dribble, A4 (Passing Integration)

1. Objectives

- **Cognitive:** Understand the concept of zigzag dribbling for evading defenders and recall passing options.
- **Skill:** Perform controlled zigzag dribble through cones and integrate with chest/bounce/overhead passes.
- **Physical:** Improve agility, speed, and coordination in direction change.
- **Affective:** Encourage persistence under pressure and cooperative play.

2. Lesson Phases

A. Warm-Up (10 minutes)

- Jogging with ball, including sudden direction changes.
- Dynamic stretches with ball (side lunges, torso twists).
- Quick partner passing in motion.

B. Physical Preparation (10 minutes)

- Cone shuffle runs without ball.
- Agility ladder drills with ball touches.

C. Main Skill Development (50 minutes) – Reciprocal Style

- Students paired (Performer + Observer).
- Teacher provides observation checklists.
- Performers execute, observers evaluate, then switch roles.

Skills & Drills:

- **A5b-D01: Zigzag Cones Dribble** – Dribble through cone line with controlled changes.
- **A5b-D02: Dribble with Defender Pressure** – Perform zigzag dribble while partner adds light defensive pressure.
- **A5b-D03: Speed Zigzag Race** – Time-based zigzag dribble race.
- **A4b-D02: Bounce Pass on Move** – Combine dribble with bounce pass while moving.
- **A4c-D02: Overhead Pass on Move** – Combine zigzag dribble with overhead pass finish.

D. Application Game (15 minutes)

- "Zigzag & Pass Game": Teams must zigzag dribble through cones, then complete an accurate pass to score a point.

E. Cool-Down (5 minutes)

- Gentle jogging, static stretches for legs and shoulders.
- Partner passing at slow pace.

3. Reciprocal Style Implementation

- **Performer:** Executes zigzag dribble and integrated passes.
- **Observer:** Monitors body posture, ball control, and accuracy of passes.
- **Feedback:** Immediate peer comments: positive reinforcement + one area to improve.
- **Switch Roles:** After each drill (5–7 minutes).
- **Teacher's Role:** Supervise, provide technical cues, ensure fair peer feedback.

4. Observation Checklist

Skill Code	Drill	Key Performance Criteria	Observer Notes
A5b-D01	Zigzag Cones Dribble	• Dribbles close to cones<br>• Maintains low stance<br>• Eyes forward	
A5b-D02	Dribble with Defender Pressure	• Protects ball<br>• Quick change of direction<br>• Maintains control	
A5b-D03	Speed Zigzag Race	• Completes zigzag quickly<br>• Maintains control at speed<br>• No ball loss	
A4b-D02	Bounce Pass on Move	• Executes bounce at right distance<br>• Accurate to partner<br>• Maintains movement speed	
A4c-D02	Overhead Pass on Move	• Releases above head<br>• Accurate long pass<br>• Maintains balance	

Teaching Unit 9 – Protective Dribble & Catch/Pass Integration

Date: 9 Apr 2025
Duration: 90 minutes
Focus Skills: A5c – Protective Dribble, Integration with A3 (Catching) & A4 (Passing)

1. Objectives

- **Cognitive:** Recognize when and how to use protective dribble against a defender.
- **Skill:** Perform protective dribble under pressure and combine with catch–pass sequences.
- **Physical:** Enhance balance, strength, and stability under contact.
- **Affective:** Build composure under defensive pressure and cooperation with teammates.

2. Lesson Phases

A. Warm-Up (10 minutes)

- Jogging with ball, including sudden stops and turns.
- Dynamic stretches with ball shielding motions.
- Partner rolling challenge: keep ball away while jogging.

B. Physical Preparation (10 minutes)

- Wall-sit with ball protection (holding ball close while seated).
- Partner resistance runs (light push on shoulder while dribbling).

C. Main Skill Development (50 minutes) – Reciprocal Style

- Students work in pairs (Performer + Observer).
- Teacher distributes observation task cards.
- Performers execute drills, observers record, then switch roles.

Skills & Drills:

- **A5c-D01: Shielded Dribble Static** – Dribble in place while shielding ball with body.
- **A5c-D02: Dribble with Defender Contact** – Maintain dribble while partner applies light shoulder pressure.
- **A3a-D02: Catch in Pairs** – Combine catch after partner's protective dribble.
- **A4b-D01: Bounce Pass Static** – Bounce pass after protecting ball.

D. Application Game (15 minutes)

- "Protect & Pass Game": One attacker protects ball for 5 seconds, then passes to teammate. Defender applies moderate pressure.

E. Cool-Down (5 minutes)

- Stretching focusing on lower back, legs, and shoulders.
- Gentle ball handling to relax.

3. Reciprocal Style Implementation

- **Performer:** Executes protective dribble, catch, and pass.
- **Observer:** Assesses shielding technique, control, and accuracy.
- **Feedback:** Observer provides quick positive note + one improvement point.
- **Switch Roles:** After each drill.
- **Teacher's Role:** Oversee feedback, ensure safety in contact drills, reinforce technical accuracy.

4. Observation Checklist

Skill Code	Drill	Key Performance Criteria	Observer Notes
A5c-D01	Shielded Dribble Static	• Body between defender & ball<br>• Low dribble<br>• Maintains balance	
A5c-D02	Dribble with Defender Contact	• Shields with shoulder/hip<br>• Maintains control<br>• Eyes forward	
A3a-D02	Catch in Pairs	• Shows hands early<br>• Absorbs ball to chest<br>• Balance maintained	
A4b-D01	Bounce Pass Static	• Bounce at ⅔ distance<br>• Accurate to partner<br>• Proper step forward	

Teaching Unit 10 – Speed Dribble & Overarm Shot

Date: 16 Apr 2025
Duration: 90 minutes
Focus Skills: A5d – Speed Dribble, A6a – Overarm Shot

1. Objectives

- **Cognitive:** Identify when to use speed dribble in fast breaks and the technical cues for overarm shooting.
- **Skill:** Perform controlled sprint dribble and execute stationary overarm shot with accuracy.
- **Physical:** Improve sprint speed, arm strength, and shooting coordination.
- **Affective:** Build confidence in attacking situations and self-efficacy in scoring.

2. Lesson Phases

A. Warm-Up (10 minutes)

- Jogging and sprinting with ball at intervals.
- Dynamic stretches with ball (arm swings, trunk twists).
- Partner passing while jogging.

B. Physical Preparation (10 minutes)

- Sprint drills: 20–30 m sprints while dribbling.
- Reaction sprint starts with ball.

C. Main Skill Development (50 minutes) – Reciprocal Style

- Students paired: one **Performer**, one **Observer**.
- Teacher provides task cards with clear criteria.
- Performers execute drills, observers evaluate and give feedback.
- Switch roles every 5–7 minutes.

Skills & Drills:

- **A5d-D01: Sprint Dribble Solo** – Dribble at maximum speed in straight line, keep ball close.
- **A5d-D02: Fast Break Dribble with Finish** – Sprint dribble and finish with pass or shot.
- **A6a-D01: Overarm Shot Static** – Stationary overarm shot at goal, focus on technique.
- **A6a-D02: Overarm Shot with Step** – Add step forward for power and accuracy.

D. Application Game (15 minutes)

- “Fast Break Challenge”: Teams dribble full court at speed and finish with overarm shot.

E. Cool-Down (5 minutes)

- Gentle jogging, static stretching for shoulders, arms, and calves.
- Partner relaxed passes.

3. Reciprocal Style Implementation

- **Performer:** Executes sprint dribble or overarm shot.
- **Observer:** Uses checklist to monitor speed, control, and shot technique.
- **Feedback:** Observer provides immediate supportive feedback (positive + one correction).
- **Switch Roles:** After each drill.
- **Teacher’s Role:** Guide peer observation, ensure sprint safety, reinforce correct shooting mechanics.

4. Observation Checklist

Skill Code	Drill	Key Performance Criteria	Observer Notes
A5d-D01	Sprint Dribble Solo	• Ball kept near body<br>• Sprint speed maintained<br>• Eyes forward	
A5d-D02	Fast Break Dribble with Finish	• Controls ball at high speed<br>• Executes finish (pass/shot)<br>• Maintains balance	
A6a-D01	Overarm Shot Static	• Elbow raised above shoulder<br>• Ball released high<br>• Follow-through applied	
A6a-D02	Overarm Shot with Step	• Step with opposite foot<br>• Strong release<br>• Accurate shot to target	

Teaching Unit 11 – Spin Dribble & Passing Fake

Date: 23 Apr 2025
Duration: 90 minutes
Focus Skills: A5e – Spin/Reverse Dribble, A7a – Passing Fake

1. Objectives

- **Cognitive:** Recognize when to use spin dribble to evade defenders and how to apply passing fakes.
- **Skill:** Perform controlled spin dribble and execute realistic chest-pass fakes.
- **Physical:** Improve agility, reaction speed, and balance under defensive pressure.
- **Affective:** Build creativity, assertiveness, and confidence in deceptive play.

2. Lesson Phases

A. Warm-Up (10 minutes)

- Jogging with ball, add 360° turns while dribbling.
- Dynamic stretches focusing on hips, torso, and shoulders.
- Quick passing in pairs at jogging pace.

B. Physical Preparation (10 minutes)

- Cone spin drill without ball (fast pivots, 180° and 360° turns).
- Sprint-stop-spin with ball control.

C. Main Skill Development (50 minutes) – Reciprocal Style

- Students paired (Performer + Observer).
- Teacher provides task cards with criteria.
- Performers execute, observers assess, then switch roles.

Skills & Drills:

- **A5e-D01: Spin Dribble Static** – Dribble, pivot, and spin while keeping ball in control.
- **A5e-D02: Spin Dribble vs Defender** – Perform spin dribble while partner applies light defence.
- **A7a-D01: Chest Pass Fake Static** – Perform chest pass fake with convincing motion.
- **A7a-D02: Fake then Real Pass** – Fake chest pass then deliver actual pass to teammate.

D. Application Game (15 minutes)

- “Deception Game”: Players must use at least one spin dribble or fake pass before completing a scoring pass.

E. Cool-Down (5 minutes)

- Gentle jogging with ball, static stretches for hips and shoulders.
- Partner relaxed passing.

3. Reciprocal Style Implementation

- **Performer:** Executes spin dribble or passing fake.
- **Observer:** Uses checklist to monitor control, realism, and decision-making.
- **Feedback:** Immediate peer-to-peer feedback (positive + correction).
- **Switch Roles:** After each drill.
- **Teacher’s Role:** Reinforce deceptive play mechanics, monitor accuracy of peer assessment.

4. Observation Checklist

Skill Code	Drill	Key Performance Criteria	Observer Notes
A5e-D01	Spin Dribble Static	• Pivots smoothly<br>• Maintains ball control<br>• Eyes forward	
A5e-D02	Spin Dribble vs Defender	• Protects ball during spin<br>• Quick hand change<br>• Maintains balance	
A7a-D01	Chest Pass Fake Static	• Convincing pass motion<br>• Maintains grip<br>• Eyes directed toward target	
A7a-D02	Fake then Real Pass	• Fake realistic<br>• Quick transition to real pass<br>• Accurate delivery	

Teaching Unit 12 – Lob Shot, Bounce Shot & Shot Fake

Date: 30 Apr 2025
Duration: 90 minutes
Focus Skills: A6b – Lob Shot, A6c – Bounce Shot, A7b – Shot Fake

1. Objectives

- **Cognitive:** Recognize situations for lob and bounce shots, and understand the purpose of shot fakes.
- **Skill:** Perform lob and bounce shots with accuracy, and execute realistic shot fakes.
- **Physical:** Enhance arm power, coordination, and trunk control.
- **Affective:** Build confidence in shooting under pressure and creativity in deception.

2. Lesson Phases

A. Warm-Up (10 minutes)

- Jogging with ball, include high-knee skips.
- Dynamic stretches focusing on shoulders and trunk.
- Partner light passing, alternating chest and overhead passes.

B. Physical Preparation (10 minutes)

- Medicine ball overhead throws for arm strength.
- Quick jump squats while holding ball.

C. Main Skill Development (50 minutes) – Reciprocal Style

- Students paired (Performer + Observer).
- Teacher distributes observation checklists with performance criteria.
- Performers execute, observers record, then switch roles.

Skills & Drills:

- **A6b-D01: Lob Shot Static** – Stationary lob shot over partner with raised arms.
- **A6b-D02: Lob Shot vs Defender** – Shoot lob over defender’s block.
- **A6c-D01: Bounce Shot Solo** – Bounce shot to goal, aiming for bottom corners.
- **A6c-D02: Bounce Shot in Pairs** – One shoots bounce, partner receives/repeats.
- **A7b-D01: Overarm Shot Fake** – Simulate overarm shot, hold, then switch action.
- **A7b-D02: Fake then Dribble/Shot** – Fake shot, then immediately dribble or shoot.

D. Application Game (15 minutes)

- "Score with Trick": Players must attempt bounce, lob, or fake before scoring in 3v3 mini-game.

E. Cool-Down (5 minutes)

- Gentle jogging, then static stretches for shoulders and legs.
- Partner relaxed passing.

3. Reciprocal Style Implementation

- **Performer:** Executes lob shot, bounce shot, or shot fake.
- **Observer:** Monitors key criteria for technique, accuracy, and deception.
- **Feedback:** Observer provides immediate reinforcement + one technical correction.
- **Switch Roles:** After each drill (5–7 minutes).
- **Teacher's Role:** Oversee peer observations, correct errors, reinforce deception and finishing skills.

4. Observation Checklist

Skill Code	Drill	Key Performance Criteria	Observer Notes
A6b-D01	Lob Shot Static	• High arc trajectory<br>• Soft wrist release<br>• Accurate to target	
A6b-D02	Lob Shot vs Defender	• Clears defender<br>• Correct timing<br>• Lands inside target zone	
A6c-D01	Bounce Shot Solo	• Ball bounces near goal<br>• Correct angle<br>• Controlled power	
A6c-D02	Bounce Shot in Pairs	• Bounce consistent<br>• Accurate placement<br>• Partner receives easily	
A7b-D01	Overarm Shot Fake	• Fake realistic<br>• Maintains grip<br>• Quick recovery	
A7b-D02	Fake then Dribble/Shot	• Fake convincing<br>• Immediate follow-up<br>• Maintains control	

Teaching Unit 13 – Rotation Shot, Body Fake & Intro to Defence

Date: 7 May 2025
Duration: 90 minutes
Focus Skills: A6d – Rotation Shot, A7c – Body Fake, D1 – Defensive Stance, D2 – Help & Rotation

1. Objectives

- **Cognitive:** Understand principles of rotation shooting, body deception, and defensive stance/rotation.
- **Skill:** Execute rotation shot and body fake, and apply basic defensive stance and help defence.
- **Physical:** Develop trunk stability, reaction speed, and lateral quickness.
- **Affective:** Build creativity in attack and responsibility in defence.

2. Lesson Phases

A. Warm-Up (10 minutes)

- Jogging with ball, add spin turns.
- Dynamic stretches: hip rotations, torso twists.
- Partner passing with quick fakes.

B. Physical Preparation (10 minutes)

- Sprint–pivot–sprint drills with ball.
- Defensive slides without ball.

C. Main Skill Development (50 minutes) – Reciprocal Style

- Students in pairs (Performer + Observer).
- Teacher provides observation sheets.
- Performers practice while observers record, then switch roles.

Skills & Drills:

- **A6d-D01: Rotation Shot Solo** – Pivot, spin, and release ball mid-rotation.
- **A6d-D02: Rotation Shot vs Defender** – Perform rotation shot under defensive pressure.
- **A7c-D01: Body Fake Static** – Use body lean to fake direction, then switch.
- **A7c-D02: Body Fake with Ball** – Perform fake with dribble or step, then attack opposite side.
- **D1-D01: On-Ball Stance Drill** – Low stance, arms wide, shuffle side-to-side.
- **D2-D01: Help Defence Drill** – Two defenders rotate to cover cutting attacker.

D. Application Game (15 minutes)

- "Attack vs Defence Challenge": One attacker must use rotation shot or fake; defender must apply stance/help principles.

E. Cool-Down (5 minutes)

- Gentle jogging, static stretches (legs, hips, shoulders).
- Partner ball fakes and light tosses.

3. Reciprocal Style Implementation

- **Performer:** Executes shot, fake, or defensive drill.
- **Observer:** Monitors criteria (balance, timing, stance, control).
- **Feedback:** Immediate constructive feedback (praise + correction).
- **Switch Roles:** Every 5–7 minutes.
- **Teacher's Role:** Supervise, ensure defensive safety, reinforce correct technique.

4. Observation Checklist

Skill Code	Drill	Key Performance Criteria	Observer Notes
A6d-D01	Rotation Shot Solo	• Spins smoothly<br>• Releases at right moment<br>• Maintains balance	
A6d-D02	Rotation Shot vs Defender	• Uses pivot effectively<br>• Ball released mid-spin<br>• Accurate to target	
A7c-D01	Body Fake Static	• Convincing body shift<br>• Quick first step<br>• Maintains balance	
A7c-D02	Body Fake with Ball	• Fake realistic<br>• Immediate opposite move<br>• Ball under control	
D1-D01	On-Ball Stance Drill	• Knees bent<br>• Arms active<br>• Quick side shuffles	
D2-D01	Help Defence Drill	• Rotates on time<br>• Communicates with teammate<br>• Anticipates cut	

Teaching Unit 14 – On-Ball Defence, Goalkeeping Basics & Small-Sided Game

Date: 14 May 2025
Duration: 90 minutes
Focus Skills: D3 – On-Ball Defence, G1 – Goalkeeper Stance, SSG-1 – Small-Sided Game (3v3)

1. Objectives

- **Cognitive:** Understand 1v1 defensive principles, goalkeeper ready stance, and application of skills in small-sided games.
- **Skill:** Apply effective on-ball defence, maintain correct goalkeeper stance, and integrate skills in a 3v3 game.
- **Physical:** Improve lateral speed, reflexes, and game endurance.
- **Affective:** Develop discipline, courage, teamwork, and competitive spirit.

2. Lesson Phases

A. Warm-Up (10 minutes)

- Jogging with changes of direction.
- Dynamic stretches for arms, legs, shoulders.
- Light 1v1 shadow defence with ball.

B. Physical Preparation (10 minutes)

- Defensive slides drill across court.
- Quick reaction jumps for goalkeeper positioning.

C. Main Skill Development (50 minutes) – Reciprocal Style

- Students paired (Performer + Observer).
- Teacher provides detailed task cards.
- Performers execute, observers monitor criteria, then switch roles.

Skills & Drills:

- **D3-D01: 1v1 Defence Drill** – Defender mirrors attacker's movements.
- **D3-D02: Mirror Shuffle Drill** – Partner leads direction, defender mirrors with quick slides.
- **G1-D01: Basic Save Drill** – Goalkeeper reacts to shots by stepping forward.
- **G1-D02: Reaction Ball Drill** – GK saves unpredictable bounces.
- **SSG-D01: 3v3 Half Court** – Apply offensive and defensive skills in mini-game.

- **SSG-D02: 3v3 Transition Game** – Teams transition quickly from defence to attack.

D. Application Game (15 minutes)

- Structured **3v3 Small-Sided Game** focusing on applying defensive stance, GK saves, and quick passing.

E. Cool-Down (5 minutes)

- Gentle jogging, stretching legs and arms.
- Partner toss and catch at slow pace.

3. Reciprocal Style Implementation

- **Performer:** Executes defensive stance, GK save, or plays in SSG.
- **Observer:** Monitors body posture, control, and decision-making.
- **Feedback:** Immediate peer feedback: positive reinforcement + technical note.
- **Switch Roles:** Every 5–7 minutes.
- **Teacher's Role:** Ensure safe defence, encourage feedback quality, manage SSG.

4. Observation Checklist

Skill Code	Drill	Key Performance Criteria	Observer Notes
D3-D01	1v1 Defence Drill	• Maintains low stance<br>• Cuts angles<br>• Eyes on attacker's torso	
D3-D02	Mirror Shuffle Drill	• Quick side steps<br>• No crossing legs<br>• Maintains balance	
G1-D01	Basic Save Drill	• Ready stance maintained<br>• Steps toward ball<br>• Hands active	
G1-D02	Reaction Ball Drill	• Reacts quickly<br>• Controls rebound<br>• Eyes on ball	
SSG-D01	3v3 Half Court	• Uses learned skills<br>• Maintains spacing<br>• Effective teamwork	
SSG-D02	3v3 Transition Game	• Quick transition<br>• Cooperation in attack/defence<br>• Applies correct decisions	

Teaching Unit 15 – Zone Defence, Advanced Goalkeeping & Final Small-Sided Game

Date: 21 May 2025
Duration: 90 minutes
Focus Skills: D7 – Zone & Switch Defence, G4 – Advanced Goalkeeping, SSG-2 – Small-Sided Game (5v5)

1. Objectives

- **Cognitive:** Understand zone defence and switching principles, advanced goalkeeper saves, and team tactics in a full small-sided game.
- **Skill:** Apply zone and switch defence, execute diving/angle GK saves, and integrate all learned skills in 5v5 play.
- **Physical:** Improve anticipation, agility, reflexes, and aerobic endurance.
- **Affective:** Build teamwork, leadership, and competitive spirit under real-game conditions.

2. Lesson Phases

A. Warm-Up (10 minutes)

- Jogging with directional changes and ball.
- Dynamic stretches focusing on trunk, arms, and legs.
- Quick ball passing in groups of 4–5.

B. Physical Preparation (10 minutes)

- Defensive slides with quick rotations.
- Goalkeeper angle-positioning runs.

C. Main Skill Development (50 minutes) – Reciprocal Style

- Students in groups (Performer + Observer).
- Teacher provides observation cards with criteria for each role.
- Performers apply defence, GK saves, or game actions; observers monitor and give feedback.
- Roles rotate every 5–7 minutes.

Skills & Drills:

- **D7-D01: Zone Drill** – Defenders cover zones in half court, communicate and switch on screens.
- **D7-D02: Switch Drill** – Two defenders switch to cover attackers after screen.

- **G4-D01: Angle Save Drill** – GK cuts down shooting angle and makes save.
- **G4-D02: Diving Save Drill** – GK performs lateral diving save against wing shots.
- **SSG-D03: 5v5 Half Court** – Full small-sided game emphasizing skills integration.
- **SSG-D04: 5v5 Transition Game** – Continuous play with quick transitions between attack and defence.

D. Application Game (15 minutes)

- Structured **5v5 Small-Sided Game** with emphasis on teamwork, communication, and applying all skills (passing, catching, dribbling, shooting, faking, defence, GK).

E. Cool-Down (5 minutes)

- Light jogging, static stretching (legs, back, arms).
- Group reflection: quick discussion on what skills improved most.

3. Reciprocal Style Implementation

- **Performer:** Executes defence, goalkeeping, or plays in SSG.
- **Observer:** Uses checklist to evaluate accuracy, teamwork, and decision-making.
- **Feedback:** Observer gives constructive peer feedback (positive + correction).
- **Switch Roles:** After each drill or game sequence.
- **Teacher's Role:** Oversee peer assessment, ensure balanced play, highlight tactical understanding.

4. Observation Checklist

Skill Code	Drill	Key Performance Criteria	Observer Notes
D7-D01	Zone Drill	• Maintains zone coverage<br>• Communicates effectively<br>• Anticipates attacker movement	
D7-D02	Switch Drill	• Switches on time<br>• No defensive gaps<br>• Maintains balance	
G4-D01	Angle Save Drill	• Cuts angle correctly<br>• Quick step forward<br>• Controls rebound	
G4-D02	Diving Save Drill	• Times dive accurately<br>• Extends arms fully<br>• Recovers quickly	
SSG-D03	5v5 Half Court	• Uses all learned skills<br>• Maintains spacing<br>• Effective teamwork	

Skill Code	Drill	Key Performance Criteria	Observer Notes
SSG-D04	5v5 Transition Game	• Quick transition play<br>• Cooperative attack/defence<br>• Good decision-making	
